# Targeting apolipoprotein E and N-terminal amyloid β-protein precursor interaction improves cognition and reduces amyloid pathology in Alzheimer’s mice

**DOI:** 10.1016/j.jbc.2023.104846

**Published:** 2023-05-19

**Authors:** Darrell Sawmiller, Naoki Koyama, Masakazu Fujiwara, Tatsuya Segawa, Masahiro Maeda, Takashi Mori

**Affiliations:** 1 Department of Neurosurgery and Brain Repair, Center of Excellence for Aging and Brain Repair, Morsani College of Medicine, University of South Florida, Tampa, Florida, USA; 2 Department of Biomedical Sciences, Saitama Medical Center and University, Kawagoe, Saitama, Japan; 3 Immuno-Biological Laboratories Co, Ltd, Fujioka, Gunma, Japan; 4 Department of Pathology, Saitama Medical Center and University, Kawagoe, Saitama, Japan

**Keywords:** Alzheimer’s disease, amyloid β-protein, amyloid β-protein precursor, apolipoprotein E, low-density lipoprotein receptor, isoform

## Abstract

Apolipoprotein E (apoE) interaction with amyloid β-protein precursor (APP) has garnered attention as the therapeutic target for Alzheimer’s disease (AD). Having discovered the apoE antagonist (6KApoEp) that blocks apoE binding to N-terminal APP, we tested the therapeutic potential of 6KApoEp on AD-relevant phenotypes in amyloid β-protein precursor/presenilin 1 (APP/PS1) mice that express each human apoE isoform of apoE2, apoE3, or apoE4 (designated APP/PS1/E2, APP/PS1/E3, or APP/PS1/E4 mice). At 12 months of age, we intraperitoneally administered 6KApoEp (250 μg/kg) or vehicle once daily for 3 months. At 15 months of age, blockage of apoE and N-terminal APP interaction by 6KApoEp treatment improved cognitive impairment in most tests of learning and memory, including novel object recognition and maze tasks in APP/PS1/E2, APP/PS1/E3, and APP/PS1/E4 mice *versus* each vehicle-treated mouse line and did not alter behavior in nontransgenic littermates. Moreover, 6KApoEp therapy ameliorated brain parenchymal and cerebral vascular β-amyloid deposits and decreased abundance of amyloid β-protein (Aβ) in APP/PS1/E2, APP/PS1/E3, and APP/PS1/E4 mice *versus* each vehicle-treated mouse group. Notably, the highest effect in Aβ-lowering by 6KApoEp treatment was observed in APP/PS1/E4 mice *versus* APP/PS1/E2 or APP/PS1/E3 mice. These effects occured through shifting toward lessened amyloidogenic APP processing due to decreasing APP abundance at the plasma membrane, reducing *APP* transcription, and inhibiting p44/42 mitogen-activated protein kinase phosphorylation. Our findings provide the preclinical evidence that 6KApoEp therapy aimed at targeting apoE and N-terminal APP interaction is a promising strategy and may be suitable for patients with AD carrying the apoE4 isoform.

Alzheimer’s disease (AD) has become a worldwide public health concern. AD irreversibly affects memory and cognition in the elderly, starting with brain changes decades prior to symptoms of dementia. To date, the consensus is that AD etiology is expressed as a combination of genetic factors, amyloid β-protein (Aβ) accumulation, aberrant tau protein phosphorylation, neuroinflammation, oxidative stress, mitochondrial dysfunction, synaptic damage, and neuronal degeneration, which conspires in the onset and progression of the disease (1).

Apolipoprotein E (apoE), a constituent of plasma lipoproteins, is a lipid and cholesterol transporter (2). Specifically, apoE plays a crucial role in the mobilization and repartitioning of cholesterol and phospholipids during membrane remodeling, repair, and regeneration (3). In humans, three isoforms (*i.e.*, apoE2, apoE3, or apoE4) are encoded by three alleles (*i.e.*, ε2, ε3, or ε4) at a single apoE gene (*APOE*) locus on the long arm of human chromosome 19. The ε4 allele of *APOE* is a well-established risk factor for the onset of sporadic AD, increasing risk three to fivefold with one copy and more than 10-fold with two copies (4). The ε3 allele of *APOE* is the most frequent form and does not alter one's risk for underlying AD. Moreover, the less frequent ε2 allele of *APOE* appears to reduce the relative risk for the occurrence of AD (5). Among the gene products encoded by each allele of *APOE*, the apoE3 isoform differs from apoE2 and apoE4 isoforms by single amino acid, resulting in a commonly occurring genetic polymorphism. Specifically, the apoE3 isoform has cysteine at amino acid position 112 and arginine at 158; the apoE2 isoform has cysteine at both 112 and 158; and the apoE4 isoform has arginine at both sites (2).

Previously, apoE has been shown to co-localize by immunohistochemistry both in the extracellular senile plaques and cerebral vessel β-amyloid deposits associated with AD (6, 7, 8) as well as directly interact with Aβ (9). Moreover, it has been reported that apoE (residues 1–191) physically binds to the N-terminal region of amyloid β-protein precursor (APP) and effectively increases the amyloidogenic APP processing by enhancing intracellular APP endocytosis, thereby increasing Aβ production (10), with a rank order of potency of apoE4 > apoE3 > apoE2 (11).

Based on these reports, we have previously investigated apoE interaction with N-terminal APP as a therapeutic target for AD and identified the binding site on apoE to N-terminal APP as corresponding to the low-density lipoprotein receptor (LDLR) binding domain (residues 133–152). Moreover, we developed an antagonizing peptide to block this interaction with the addition of six lysine (K) residues to the identified 133 to 152 residues on apoE (designated as 6KApoEp) (12).

Having discovered a designer peptide that directly halts apoE binding to N-terminal APP and markedly limits apoE-mediated Aβ production (12), we tested whether 6KApoEp therapy for 3 months modifies AD-relevant phenotypes in amyloid β-protein precursor/presenilin 1 (APP/PS1) mice that express each human apoE isoform of apoE2, apoE3, or apoE4 under the endogenous regulatory control (designated as APP/PS1/E2, APP/PS1/E3, or APP/PS1/E4 mice; alternatively designated as APP/PS1/E2/E3/E4 mice). Moreover, given that both apoE3 and apoE4 have much stronger binding affinity to their receptors *versus* apoE2 (13), we further focused on the apoE isoform-specific effect in these mice after 6KApoEp therapy across AD-relevant phenotypes.

## Results

### Blockage of apoE interaction with N-terminal APP by 6KApoEp reverses learning and memory impairment

APP/PS1 mice that express each human apoE isoform have transgene-associated behavioral impairment as early as 5 to 7 months of age, so we began by assessing baseline cognitive status prior to treating at 12 months. Our cohort of 12-month-old APP/PS1/E2/E3/E4 mice *versus* wild-type (WT) littermate controls showed behavioral impairment in novel object recognition, Y-maze, and radial arm water maze (RAWM) tests (data not shown). Subsequently, we randomly assigned behaviorally impaired APP/PS1/E2/E3/E4 mice and WT littermate controls to treatment with either 6KApoEp or vehicle (*n* = 8 per group; equal numbers of four for each sex). Intraperitoneal treatment was given once daily for 3 months with 6KApoEp (250 μg/kg in 50 μl of physiological saline) or vehicle (50 μl of physiological saline) beginning at 12 months. At the end of treatment (15 months of age), we performed the same behavioral testing battery again.

**Figure 1:**
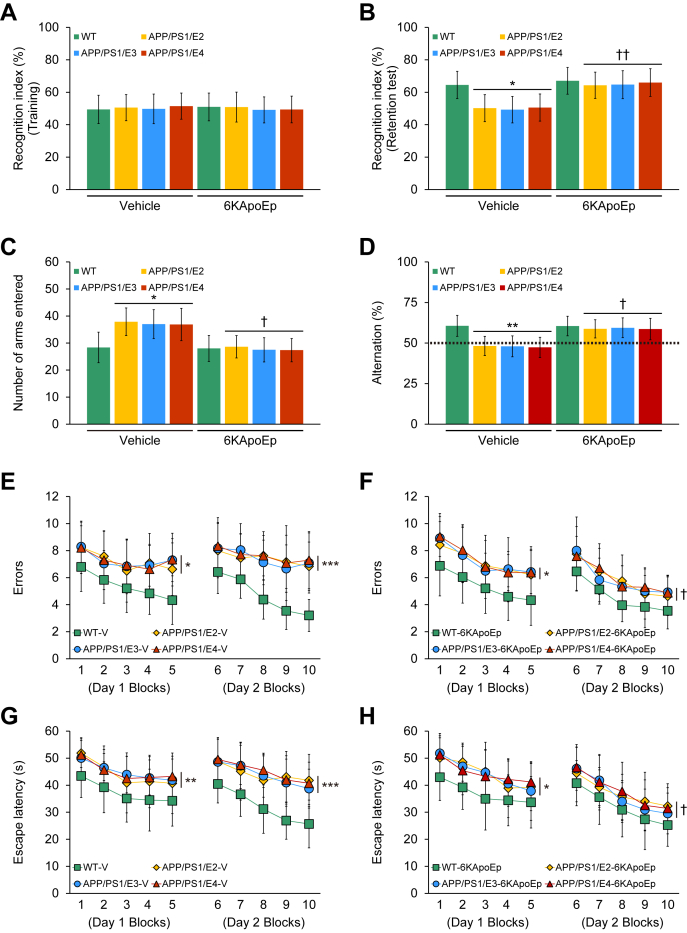
**Transgene-associated behavioral impairment completely reverses by 3-month 6KApoEp treatment**. All mice were tested in a comprehensive behavioral battery at 15 months. *A* and *B*, recognition index (%) in the novel object recognition test is shown for training (*A*) and retention (*B*) phases. *C* and *D*, Y-maze test data are represented as total arm entries (*C*) and spontaneous alternation (*D*). *E*–*H*, two-day radial arm water maze test data are shown with five blocks per day for errors (*E* and *F*) and for escape latency (*G* and *H*). Data were obtained from APP/PS1/E2 mice that received vehicle (*APP/PS1/E2-V*) or 6KApoEp (*APP/PS1/E2-6KApoEp*), APP/PS1/E3 mice that received vehicle (*APP/PS1/E3-V*) or 6KApoEp (*APP/PS1/E3-6KApoEp*), and APP/PS1/E4 mice that received vehicle (*APP/PS1/E4-V*) or 6KApoEp (*APP/PS1/E4-6KApoEp*) as well as wild-type mice that received vehicle (*WT-V*) or 6KApoEp (*WT-6KApoEp*) for 3 months after initial behavioral testing at 12 months of age. Behavioral data for (*A*–*H*) included each mouse, and measured data were averaged (*n* = 8 per group with four of each sex). Statistical comparisons for (*A*–*H*) are between groups. ∗*p* < 0.05; ∗∗*p* < 0.01; ∗∗∗*p* < 0.001 for WT-V or -6KApoEp *versus* each APP/PS1/E2/E3/E4-V or -6KApoEp mice; ^†^*p* < 0.05; ^††^*p* < 0.01 for each 6KApoEp- *versus* each vehicle-treated APP/PS1/E2/E3/E4 mice (Tables S1–S5). *V*, vehicle.

We initially assessed episodic memory by novel object recognition test. In the training phase of the test, two-way analysis of variance (ANOVA) did not reveal any significant effects of genotype and treatment, and nor any significant interaction between them. All eight mouse groups demonstrated similar recognition indices (49.1–51.4%) (Fig. 1*A*). In the retention phase of the test, two-way ANOVA disclosed the main effects of genotype (*p* < 0.05) and treatment (*p* < 0.001), and there was no significant interaction between them. The vehicle-treated APP/PS1/E2/E3/E4 mouse groups significantly decreased recognition index *versus* the vehicle-treated WT mouse group (Fig. 1*B*; ∗*p* < 0.05), failing to acquire episodic memory. 6KApoEp-treated APP/PS1/E2/E3/E4 mice had significantly increased novel object exploration frequency by 64.3 to 66.0% *versus* each vehicle-treated APP/PS1/E2/E3/E4 mice (49.3–50.6%). Interestingly, 3-month 6KApoEp therapy completely restored episodic memory (Fig. 1*B*; ^††^*p* < 0.01 for each 6KApoEp- *versus* each vehicle-treated APP/PS1/E2/E3/E4 mice), as there were no significant differences *versus* either of the WT mouse groups (Fig. 1*B*; *p* > 0.05).

We switched to evaluate the exploratory activity and spatial working memory by the alternation Y-maze task. In the evaluation for Y-maze total arm entries, two-way ANOVA revealed the main effects of genotype (*p* < 0.05) and treatment (*p* < 0.001), and the interaction between them showed significance (*p* < 0.05). Subsequently, post hoc comparison disclosed statistically significant differences between the vehicle-treated APP/PS1/E2/E3/E4 mouse groups and the vehicle-treated WT mouse group (Fig. 1*C*; ∗*p* < 0.05). This transgene-related behavioral phenotype has been noted in this and other mouse models of cerebral amyloidosis (14, 15, 16, 17, 18, 19, 20, 21, 22), and it may mirror disinhibition caused by cortical and/or hippocampal damage (23). Notably, *APP/PS1* transgene-associated hyperactivity, often engaging as anxiety-like behavior, was fully reversed by 6KApoEp treatment (Fig. 1*C*; ^†^*p* < 0.05 for each 6KApoEp- *versus* each vehicle-treated APP/PS1/E2/E3/E4 mice). 6KApoEp treatment for 3 months totally stabilized hyperactivity, as the treatment groups did not significantly differ from either of the WT mouse groups (Fig. 1*C*; *p* > 0.05).

Mice instinctively alternate arms in the Y-maze such that they enter the three arms in sequence more often than by chance alone (50%, see *dotted line* in Fig. 1*D*); this behavioral phenotype is largely interpreted as a measure of spatial working memory (15). As per our expectation, vehicle-treated APP/PS1/E2/E3/E4 mouse behavior demonstrated less tendency to alternate *versus* WT controls. In the assessment for Y-maze spontaneous alternation, two-way ANOVA presented the main effects of genotype (*p* < 0.01) and treatment (*p* < 0.001), and there was a significant interaction between them (*p* < 0.05). Post hoc comparison revealed statistically significant differences in Y-maze spontaneous alternation between the vehicle-treated APP/PS1/E2/E3/E4 mouse groups and the vehicle-treated WT mouse group (Fig. 1*D*; ∗∗*p* < 0.01). Interestingly, 6KApoEp-treated APP/PS1/E2/E3/E4 mice revealed a significantly greater percentage of spontaneous alternation *versus* vehicle-treated APP/PS1/E2/E3/E4 mice (Fig. 1*D*; ^†^*p* < 0.05), which did not significantly differ from either of the WT mouse groups (Fig. 1*D*; *p* > 0.05). Therefore, 3-month 6KApoEp therapy fully restored defective spatial working memory in the alternation Y-maze task.

Finally, we tested hippocampus-dependent spatial reference learning and memory by the RAWM test. Most mouse groups increased learning and memory as shown by reduced errors and shortened escape latency with more trials (blocks) on days 1 and 2, whereas the vehicle-treated APP/PS1/E2/E3/E4 mouse groups did not acquire learning and memory (Fig. 1, *E*–*H*). On day 1, two-way ANOVA denoted the main effect of genotype (*p* < 0.001), and the interaction between genotype and treatment did not show significance for both errors and escape latency. Repeated-measures ANOVA followed by post hoc assessment revealed statistically significant differences in the vehicle- or 6KApoEp-treated APP/PS1/E2/E3/E4 mouse groups *versus* either of the WT mouse groups (Fig. 1, *E*–*H*; ∗*p* < 0.05; ∗∗*p* < 0.01 for both errors and escape latency). On day 2, two-way ANOVA displayed the main effects of genotype (*p* < 0.001) and treatment (*p* < 0.001), and there was a significant interaction between them (*p* < 0.05) for both errors and escape latency. Repeated-measures ANOVA followed by post hoc comparison demonstrated that errors and escape latency in vehicle-treated APP/PS1/E2/E3/E4 mice were significantly greater than those in vehicle-treated WT mice (Fig. 1, *E* and *G*; ∗∗∗*p* < 0.001 for both errors and escape latency). However, 6KApoEp-treated APP/PS1/E2/E3/E4 mice accomplished the task with significantly lessened errors and shortened escape latency *versus* each vehicle-treated APP/PS1/E2/E3/E4 mice (Fig. 1, *E*–*H*; ^†^*p* < 0.05), and their behavior did not significantly differ from either of the WT mouse groups on day 2 (Fig. 1, *F* and *H*; *p* > 0.05). There were no significant between-group differences (*p* > 0.05) in swim speed nor did we find thigmotaxis (characteristic of extreme anxiety-like behavior) in any of the mice tested. Thus, behavioral differences in the RAWM test were not due to motivational issues, locomotor impairment, or anxiety. Together, these results demonstrated that 6KApoEp therapy for 3 months wholly reduces spatial reference learning and memory impairment associated with the *APP* and *PS1* transgenes.

### ApoE antagonist (6KApoEp) mitigates cerebral amyloid pathology with the highest effect in APP/PS1/E4 mice

**Figure 2:**
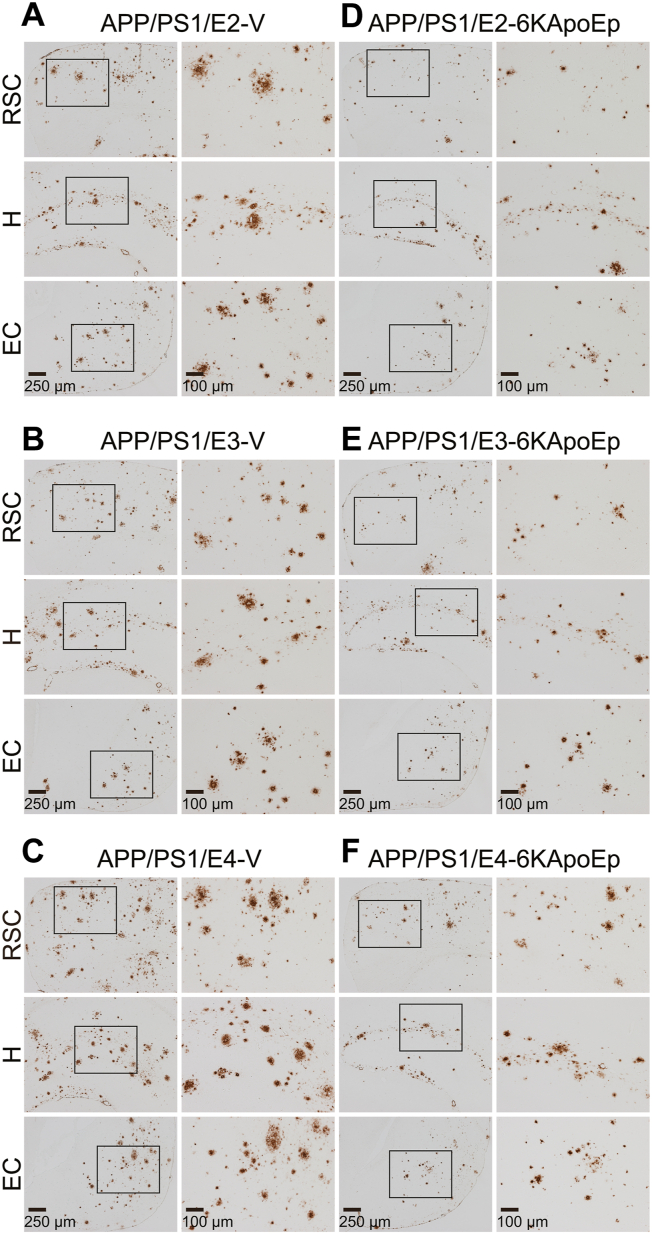
**Three-month 6KApoEp therapy reduces β-amyloid plaques in APP/PS1/E2, APP/PS1/E3, and APP/PS1/E4 mouse brains.***A*–*F*, representative images were taken from APP/PS1/E2 mice that received vehicle (*APP/PS1/E2-V*) or 6KApoEp (*APP/PS1/E2-6KApoEp*), APP/PS1/E3 mice that received vehicle (*APP/PS1/E3-V*) or 6KApoEp (*APP/PS1/E3-6KApoEp*), and APP/PS1/E4 mice that received vehicle (*APP/PS1/E4-V*) or 6KApoEp (*APP/PS1/E4-6KApoEp*) for 3 months starting at 12 months of age. Immunohistochemistry using anti-Aβ_17–24_ monoclonal antibody (*mAb 4G8*) reveals cerebral β-amyloid deposits. Brain regions shown include retrosplenial cortex (RSC, *top*); hippocampus (H, *middle*); and entorhinal cortex (EC, *bottom*). Each *right image* is a higher magnification image from *insets*. *V*, vehicle.

**Figure 3:**
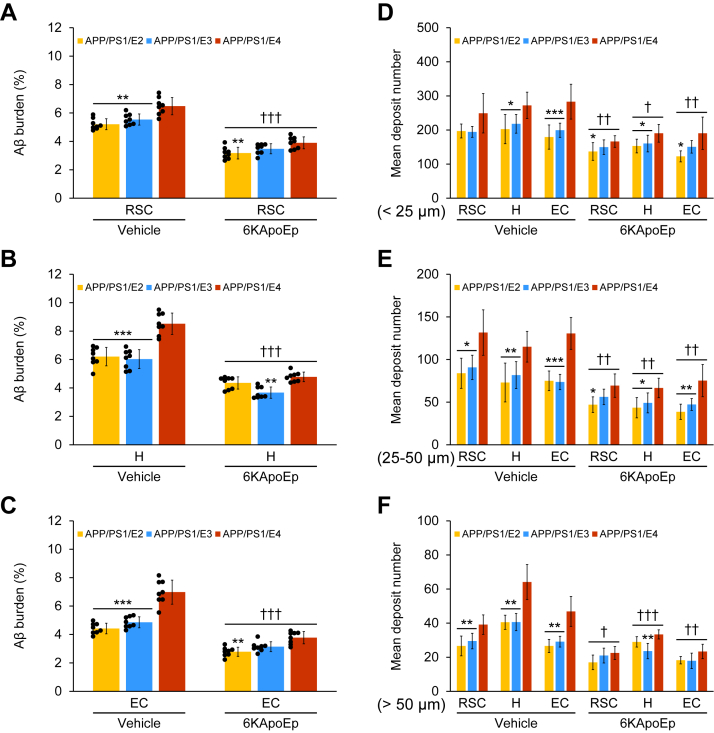
**6KApoEp treatment for 3 months effectively ameliorates cerebral parenchymal β-amyloid deposits**. *A*–*C*, quantitative image analysis of Aβ burden (%) from 4G8 immunohistochemistry is shown. *D*–*F*, morphometric analysis of cerebral parenchymal β-amyloid deposit size. Mean plaque size is shown from blind assignment to one of three mutually exclusive categories: small (<25 μm; *D*), medium (between 25 and 50 μm; *E*), or large (>50 μm; *F*). *A*–*C*, each brain region is denoted on the *x*-axis (RSC, H, and EC). *D*–*F*, mean deposit number is shown on the *y*-axis, and the brain region is listed on the *x*-axis. Data were collected from APP/PS1/E2 mice that received vehicle (*APP/PS1/E2-V*) or 6KApoEp (*APP/PS1/E2-6KApoEp*), APP/PS1/E3 mice that received vehicle (*APP/PS1/E3-V*) or 6KApoEp (*APP/PS1/E3-6KApoEp*), and APP/PS1/E4 mice that received vehicle (*APP/PS1/E4-V*) or 6KApoEp (*APP/PS1/E4-6KApoEp*) for 3 months beginning at 12 months of age. Immunohistochemistry data for (*A*–*F*) included each mouse (*n* = 8 per group with four of each sex), and quantitative data were averaged. Statistical comparisons for (*A*–*F*) are between groups within each brain region. ∗*p* < 0.05; ∗∗*p* < 0.01; ∗∗∗*p* ≤ 0.001 for APP/PS1/E2-V or APP/PS1/E3-V *versus* APP/PS1/E4-V mice, and for APP/PS1/E2-6KApoEp or APP/PS1/E3-6KApoEp *versus* APP/PS1/E4-6KApoEp mice; ^†^*p* < 0.05; ^††^*p* < 0.01; ^†††^*p* < 0.001 for each 6KApoEp- *versus* each vehicle-treated APP/PS1/E2/E3/E4 mice (Tables S6–S9). *V*, vehicle.

At 15 months of age, vehicle-treated APP/PS1/E2/E3/E4 mice had progressive cerebral amyloid pathology (average of 4–9% cerebral β-amyloid burden by anti-Aβ_17–24_ monoclonal antibody, mAb 4G8) throughout the retrosplenial cortex (RSC), hippocampus (H), and entorhinal cortex (EC) regions of interest (ROI). In the image analysis for cerebral β-amyloid burden, two-way ANOVA revealed the main effects of genotype (*p* < 0.001) and treatment (*p* < 0.001) for RSC, H, and EC, and the interaction between them showed significance (*p* < 0.001) for both H and EC. Interestingly, among the vehicle-treated mouse groups, APP/PS1/E4 mice showed a significantly higher percentage of cerebral β-amyloid burden across all three brain regions *versus* APP/PS1/E2 or APP/PS1/E3 mice (Figs. 2, *A*–*C*, and 3, *A*–*C*; ∗∗*p* < 0.01 for RSC; ∗∗∗*p* ≤ 0.001 for H and EC). 6KApoEp treatment significantly attenuated cerebral β-amyloid burden across all three brain regions: APP/PS1/E2 mice (30–39%); APP/PS1/E3 mice (35–39%); APP/PS1/E4 mice (40–46%) (Figs. 2, *D*–*F*, and 3, *A*–*C*; ^†††^*p* < 0.001 for each 6KApoEp- *versus* each vehicle-treated APP/PS1/E2/E3/E4 mice), and significant differences in *APOE* genotype disclosed less *p*-value in the 6KApoEp-treated mouse groups *versus* the vehicle-treated mouse groups across all three brain regions (Figs. 2, *D*–*F*, and 3, *A*–*C*; ∗∗*p* < 0.01 for RSC, H, and EC). Intriguingly, among the 6KApoEp-treated mouse groups, the highest effect for Aβ-lowering was revealed in APP/PS1/E4 mice across all three brain regions *versus* the other APP/PS1/E2 or APP/PS1/E3 mice (Figs. 2, *D*–*F*, and 3, *A*–*C*).

To clarify whether the attenuated cerebral β-amyloid burden was specific to a plaque subset or occurred more generally, we did morphometric analysis by blindly assigning β-amyloid plaques to one of three mutually exclusive categories based on maximum diameter: small (<25 μm); medium (between 25 and 50 μm); or large (>50 μm). In the morphometric analysis for the mean deposit number, two-way ANOVA exhibited the main effects of genotype (*p* ≤ 0.001) and treatment (*p* < 0.001) for small: RSC, H, and EC; medium: RSC, H, and EC; large: RSC, H, and EC, and there was a significant interaction between them (*p* < 0.05) for medium: RSC and EC; large: RSC, H, and EC. Notably, among the vehicle-treated mouse groups, APP/PS1/E4 mice demonstrated significant increase in the mean deposit number across most three plaque sizes and brain regions *versus* APP/PS1/E2 or APP/PS1/E3 mice (Figs. 2, *A*–*C*, and 3, *D*–*F*; ∗*p* < 0.05; ∗∗*p* < 0.01; ∗∗∗*p* ≤ 0.001). 6KApoEp therapy exerted significant reductions in the mean deposit number across all three plaque sizes and brain regions: APP/PS1/E2 mice, small (24–32%), medium (40–49%), and large (28–36%); APP/PS1/E3 mice, small (23–27%), medium (36–40%), and large (29–42%); APP/PS1/E4 mice, small (30–33%), medium (42–47%), and large (42–50%) (Figs. 2, *D*–*F*, and 3, *D*–*F*; ^†^*p* < 0.05; ^††^*p* < 0.01; ^†††^*p* < 0.001 for each 6KApoEp- *versus* each vehicle-treated APP/PS1/E2/E3/E4 mice), and there were significant differences with less *p*-value in *APOE* genotype in the 6KApoEp-treated mouse groups *versus* the vehicle-treated mouse groups across most three plaque sizes and brain regions (Figs. 2, *D*–*F*, and 3, *D*–*F*; ∗*p* < 0.05; ∗∗*p* < 0.01). Importantly, among three lines of mice, the strongest effect for reductions in the mean deposit number by 6KApoEp therapy was revealed in APP/PS1/E4 mice across most three plaque sizes and brain regions (Figs. 2, *D*–*F*, and 3, *D*–*F*).

**Figure 4:**
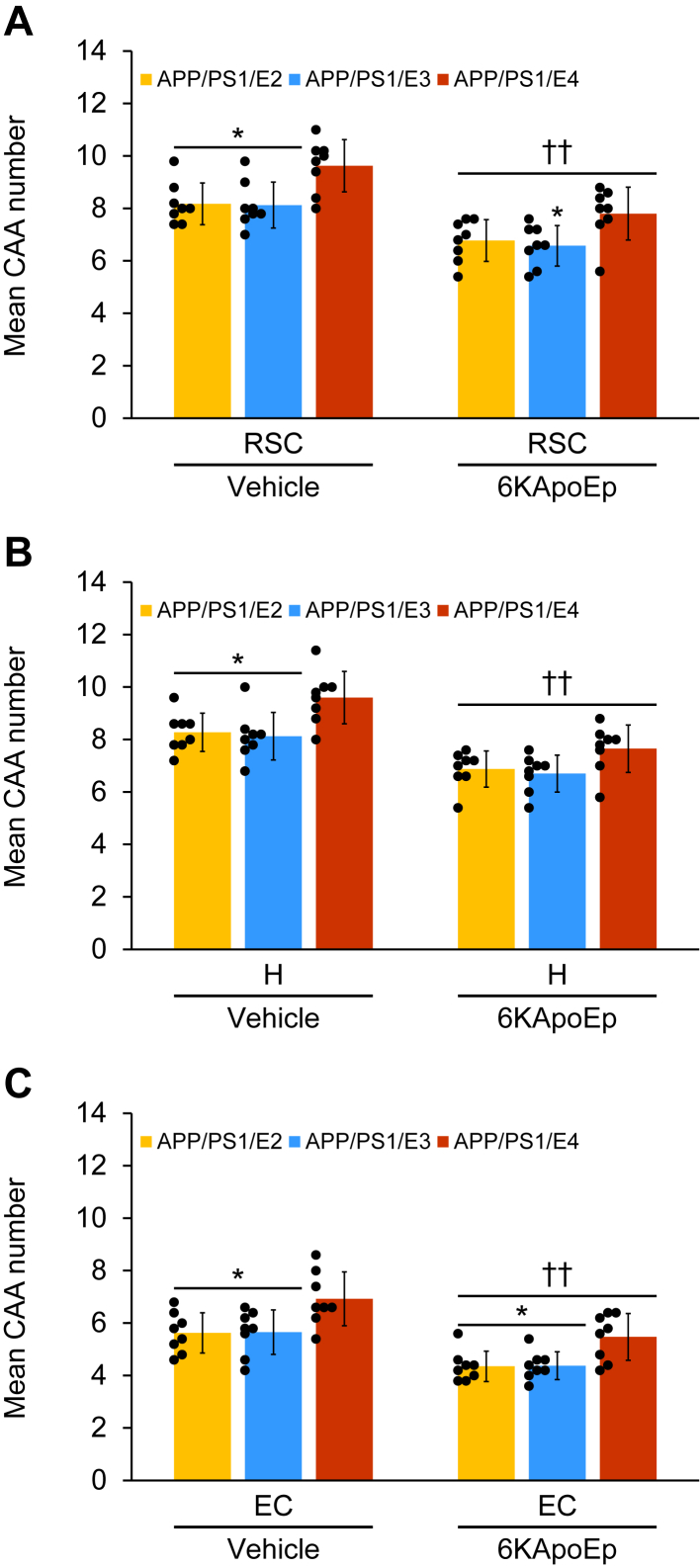
**Three-month 6KApoEp therapy effectively reduces cerebral vascular β-amyloid deposits**. *A*–*C*, severity of cerebral amyloid angiopathy (mean CAA number) is shown. The mean CAA number is shown on the *y*-axis, and the brain region is presented on the *x*-axis. Data were collected from APP/PS1/E2 mice that received vehicle (*APP/PS1/E2-V*) or 6KApoEp (*APP/PS1/E2-6KApoEp*), APP/PS1/E3 mice that received vehicle (*APP/PS1/E3-V*) or 6KApoEp (*APP/PS1/E3-6KApoEp*), and APP/PS1/E4 mice that received vehicle (*APP/PS1/E4-V*) or 6KApoEp (*APP/PS1/E4-6KApoEp*) for 3 months beginning at 12 months of age. Immunohistochemistry data for (*A*–*C*) included each mouse (*n* = 8 per group with four of each sex), and quantitative data were averaged. Statistical comparisons for (*A*–*C*) are between groups within each brain region. ∗*p* < 0.05 for APP/PS1/E2-V or APP/PS1/E3-V *versus* APP/PS1/E4-V mice, and for APP/PS1/E2-6KApoEp or APP/PS1/E3-6KApoEp *versus* APP/PS1/E4-6KApoEp mice; ^††^*p* < 0.01 for each 6KApoEp- *versus* each vehicle-treated APP/PS1/E2/E3/E4 mice (Table S10). *V*, vehicle.

As 83% of patients having AD develop cerebral amyloid angiopathy (CAA) with age, that is, vascular β-amyloid deposits (24), we also examined CAA pathology in our experimental paradigm. We blindly counted 4G8 antibody-stained cerebral vascular β-amyloid deposits in walls of penetrating arteries at the pial surface in RSC and EC areas and in small arteries at the hippocampal fissure and brachium of the superior colliculus in the hippocampal region. In the quantitative analysis for the mean CAA number, two-way ANOVA displayed the main effects of genotype (*p* < 0.001) and treatment (*p* < 0.001) for RSC, H, and EC, and the interaction between them did not show significance in all three brain regions. In the vehicle-treated mouse groups, APP/PS1/E4 mice revealed a significant increase in the mean CAA number across all three brain regions *versus* APP/PS1/E2 or APP/PS1/E3 mice (Fig. 4, *A*–*C*; ∗*p* < 0.05), which was significantly decreased by 6KApoEp therapy across all three brain regions: APP/PS1/E2 mice (17–23%); APP/PS1/E3 mice (18–23%); APP/PS1/E4 mice (19–21%) (Fig. 4, *A*–*C*; ^††^*p* < 0.01 for each 6KApoEp- *versus* each vehicle-treated APP/PS1/E2/E3/E4 mice) with less *p*-value of significant differences in *APOE* genotype in the 6KApoEp-treated mouse groups *versus* the vehicle-treated mouse groups across all three brain regions (Fig. 4, *A*–*C*; ∗*p* < 0.05).

**Figure 5:**
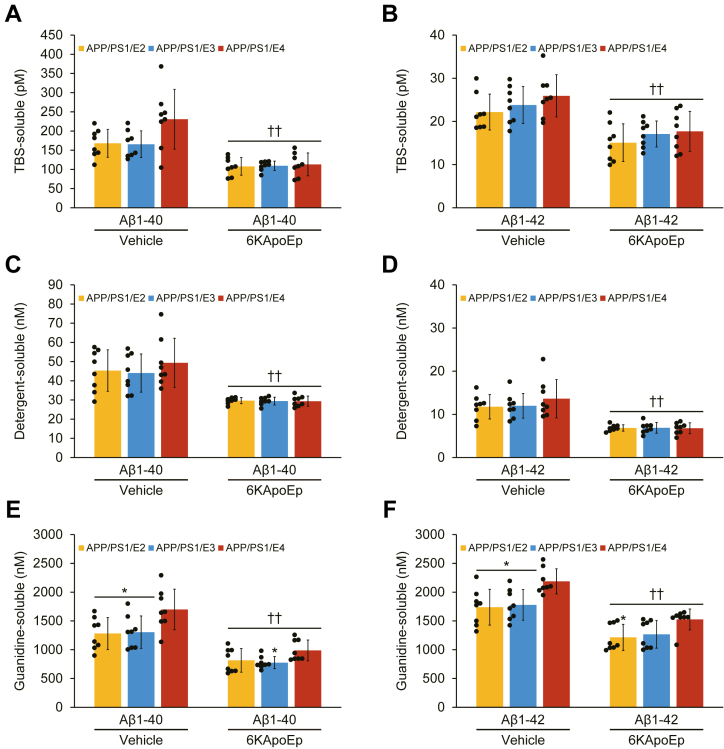
**6KApoEp treatment for 3 months effectively lowers Aβ levels.***A* and *B*, TBS-soluble; *C* and *D*, detergent-soluble; and *E* and *F*, 5 M guanidine-HCl-extractable fractions from brain homogenates were individually measured by sandwich ELISA for human Aβ_1–40_ and Aβ_1–42_. Data were collected from APP/PS1/E2 mice that received vehicle (*APP/PS1/E2-V*) or 6KApoEp (*APP/PS1/E2-6KApoEp*), APP/PS1/E3 mice that received vehicle (*APP/PS1/E3-V*) or 6KApoEp (*APP/PS1/E3-6KApoEp*), and APP/PS1/E4 mice that received vehicle (*APP/PS1/E4-V*) or 6KApoEp (*APP/PS1/E4-6KApoEp*) for 3 months beginning at 12 months of age. Sandwich ELISA data for (*A*–*F*) included each mouse (*n* = 8 per group with four of each sex), and measured data were averaged. Statistical comparisons for (*A*–*F*) are between groups for each Aβ species. ∗*p* < 0.05 for APP/PS1/E2-V or APP/PS1/E3-V *versus* APP/PS1/E4-V mice, and for APP/PS1/E2-6KApoEp or APP/PS1/E3-6KApoEp *versus* APP/PS1/E4-6KApoEp mice; ^††^*p* < 0.01 for each 6KApoEp- *versus* each vehicle-treated APP/PS1/E2/E3/E4 mice (Tables S11–S13). *V*, vehicle.

Moreover, we verified histological findings with biochemical analysis of Aβ species in brain homogenates using sandwich enzyme-linked immunosorbent assay (ELISA). In the biochemical analysis for Aβ species, two-way ANOVA disclosed the main effects of genotype (*p* < 0.05) for Aβ_1–40_ in the TBS-soluble fraction; (*p* ≤ 0.001) for both Aβ_1–40_ and Aβ_1–42_ in the guanidine-HCl-soluble pellet and treatment (*p* < 0.001) for both Aβ species across three fractions, and there was no significant interaction between them for both Aβ species across three fractions. In the TBS-soluble fraction from the vehicle-treated mouse groups, APP/PS1/E4 mice revealed increase in Aβ_1–40_ (27–28%) and Aβ_1–42_ (3–5%) *versus* APP/PS1/E2 or APP/PS1/E3 mice (Fig. 5, *A* and *B*), which did not reach significances. Moreover, 6KApoEp therapy exerted significant reductions in APP/PS1/E2 mice, Aβ_1–40_ (36%) and Aβ_1–42_ (32%); APP/PS1/E3 mice, Aβ_1–40_ (34%) and Aβ_1–42_ (28%); APP/PS1/E4 mice, Aβ_1–40_ (51%) and Aβ_1–42_ (32%) (Fig. 5, *A* and *B*; ^††^*p* < 0.01 for each 6KApoEp- *versus* each vehicle-treated APP/PS1/E2/E3/E4 mice). A similar trend was noted when considering the detergent-soluble fraction, where vehicle-treated APP/PS1/E4 mice showed increase in Aβ_1–40_ (8–11%) and Aβ_1–42_ (12–14%) (Fig. 5, *C* and *D*) *versus* vehicle-treated APP/PS1/E2 or APP/PS1/E3 mice with significant reductions in Aβ_1–40_ (33–40%) and Aβ_1–42_ (42–50%) by 6KApoEp therapy (Fig. 5, *C* and *D*; ^††^*p* < 0.01 for both Aβ_1–40_ and Aβ_1–42_). Finally, in the guanidine-HCl-soluble pellet, which most closely mirrors β-amyloid deposits, APP/PS1/E4 mice disclosed significantly high levels for Aβ_1–40_ (23–25%) and Aβ_1–42_ (19–21%) *versus* APP/PS1/E2 or APP/PS1/E3 mice in the vehicle-treated mouse groups (Fig. 5, *E* and *F*; ∗*p* < 0.05 for both Aβ_1–40_ and Aβ_1–42_) with marked reductions in 6KApoEp-treated APP/PS1/E2/E3/E4 mouse groups for Aβ_1–40_ (36–42%) and Aβ_1–42_ (29–30%) (Fig. 5, *E* and *F*; ^††^*p* < 0.01 for both Aβ_1–40_ and Aβ_1–42_). Moreover, significant differences in *APOE* genotype noted less *p*-value in the 6KApoEp-treated mouse groups *versus* the vehicle-treated mouse groups in both Aβ_1–40_ and Aβ_1–42_ species (Fig. 5, *E* and *F*; ∗*p* < 0.05). Here, among three lines of APP/PS1/E2/E3/E4 mice, the strongest effect on reducing Aβ_1–40_ and Aβ_1–42_ abundance by 6KApoEp therapy was demonstrated in APP/PS1/E4 mice across most three fractions (Fig. 5, *A*–*F*).

### Decrease in APP abundance at the plasma membrane, attenuation of APP transcription, and reduction of amyloidogenic APP processing by 6KApoEp therapy

**Figure 6:**
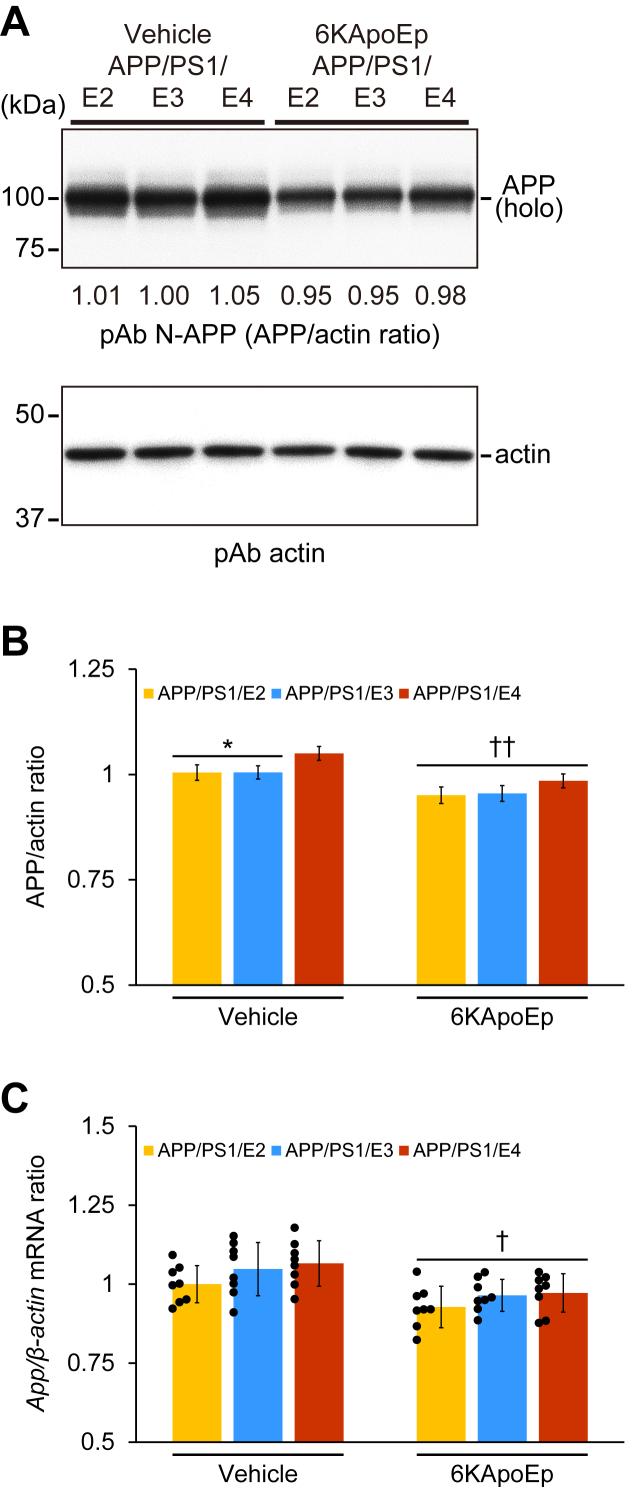
**6KApoEp therapy reduces protein expression of APP at the plasma membrane and decreases brain *APP* mRNA level**. *A*, Western blots are shown using anti-N-terminal APP polyclonal antibody (*pAb N-APP*). Actin is included as a loading control, and densitometry values are indicated *below* each lane. Equal amounts of total protein were loaded per lane. *B*, densitometry data are shown for ratios of APP to actin. *C*, QPCR for *App*. *β-actin* is used as an internal reference control. Data for (*C*) is expressed as a relative fold over APP/PS1/E2-V mice. Data were obtained from APP/PS1/E2 mice that received vehicle (*APP/PS1/E2-V*) or 6KApoEp (*APP/PS1/E2-6KApoEp*), APP/PS1/E3 mice that received vehicle (*APP/PS1/E3-V*) or 6KApoEp (*APP/PS1/E3-6KApoEp*), and APP/PS1/E4 mice that received vehicle (*APP/PS1/E4-V*) or 6KApoEp (*APP/PS1/E4-6KApoEp*) for 3 months starting at 12 months of age. Western blotting data for (*B*) included each mouse (*n* = 4 per group with two of each sex), and measured data were averaged. QPCR for (*C*) included each mouse (*n* = 8 per group with four of each sex), and quantitative data were averaged. Statistical comparisons for (*B* and *C*) are between groups. ∗*p* < 0.05 for APP/PS1/E2-V or APP/PS1/E3-V *versus* APP/PS1/E4-V mice; ^†^*p* < 0.05; ^††^*p* < 0.01 for each 6KApoEp- *versus* each vehicle-treated APP/PS1/E2/E3/E4 mice (Tables S14 and S15). *V*, vehicle.

Physiologically, APP is synthesized in the endoplasmic reticulum and trafficked *via* the *trans*-Golgi network to the plasma membrane, where the majority (90%) of APP molecules are cleaved *via* nonamyloidogenic α-secrease (25, 26), and the remaining unprocessed APP (∼10%) is cleaved at the plasma membrane or further trafficked back into the cell by endocytosis, followed by cleavage by amyloidogenic β-site APP−cleaving enzyme 1 (BACE1, β-secretase); therefore, Aβ species are constitutively generated at low levels. Sequential amyloidogenic APP endoproteolysis by the β- and γ-secretases generates β-C-terminal APP fragment (β-CTF/C99) and soluble APP-β followed by Aβ, whereas the nonamyloidogenic pathway generates α-C-terminal APP fragment (α-CTF/C83) and soluble APP-α (27, 28, 29, 30). We hypothesized that amelioration of cerebral amyloidosis in 6KApoEp-treated APP/PS1/E2/E3/E4 mouse groups might be due to reducing APP abundance at the plasma membrane by inhibition of APP trafficking to the plasma membrane as observed recently (12). In order to confirm these findings, we assessed APP expression at the plasma membrane by Western blotting using the detergent-soluble fraction of brain homogenates. We verified that the detergent-soluble fraction contains primarily plasma membrane proteins and less cytosol component proteins by Western blotting of the plasma membrane protein sodium-potassium ATPase and the cytosolic protein heat-shock protein 90 (data not shown). In the densitometry analysis for the plasma membrane APP, two-way ANOVA disclosed the main effects of genotype (*p* < 0.001) and treatment (*p* < 0.001), and the interaction between them did not show significance. The band density of plasma membrane APP was significantly enhanced in APP/PS1/E4 mice *versus* APP/PS1/E2 or APP/PS1/E3 mice in the vehicle-treated mouse groups (Fig. 6, *A* and *B*; ∗*p* < 0.05). In addition, 6KApoEp treatment for 3 months significantly decreased the band density of plasma membrane APP in APP/PS1/E2/E3/E4 mice (Fig. 6, *A* and *B*; ^††^*p* < 0.01 for each 6KApoEp- *versus* each vehicle-treated APP/PS1/E2/E3/E4 mice), and significant differences in *APOE* genotype did not denote in the 6KApoEp-treated mouse groups.

Moreover, we examined whether 6KApoEp therapy alters brain *APP* transcription, and assayed *APP* mRNA expression in vehicle- and 6KApoEp-treated APP/PS1/E2/E3/E4 mouse brains by quantitative real-time PCR (QPCR). In the quantitative analysis for brain *APP* mRNA expression, two-way ANOVA exhibited the main effect of treatment (*p* < 0.001), and the interaction between genotype and treatment did not show significance. Importantly, 6KApoEp therapy significantly decreased brain mRNA expression of *APP* in APP/PS1/E2/E3/E4 mice (Fig. 6*C*; ^†^*p* < 0.05 for each 6KApoEp- *versus* each vehicle-treated APP/PS1/E2/E3/E4 mice), and there were no significant differences in *APOE* genotype within vehicle- or 6KApoEp-treated APP/PS1/E2/E3/E4 mice.

**Figure 7:**
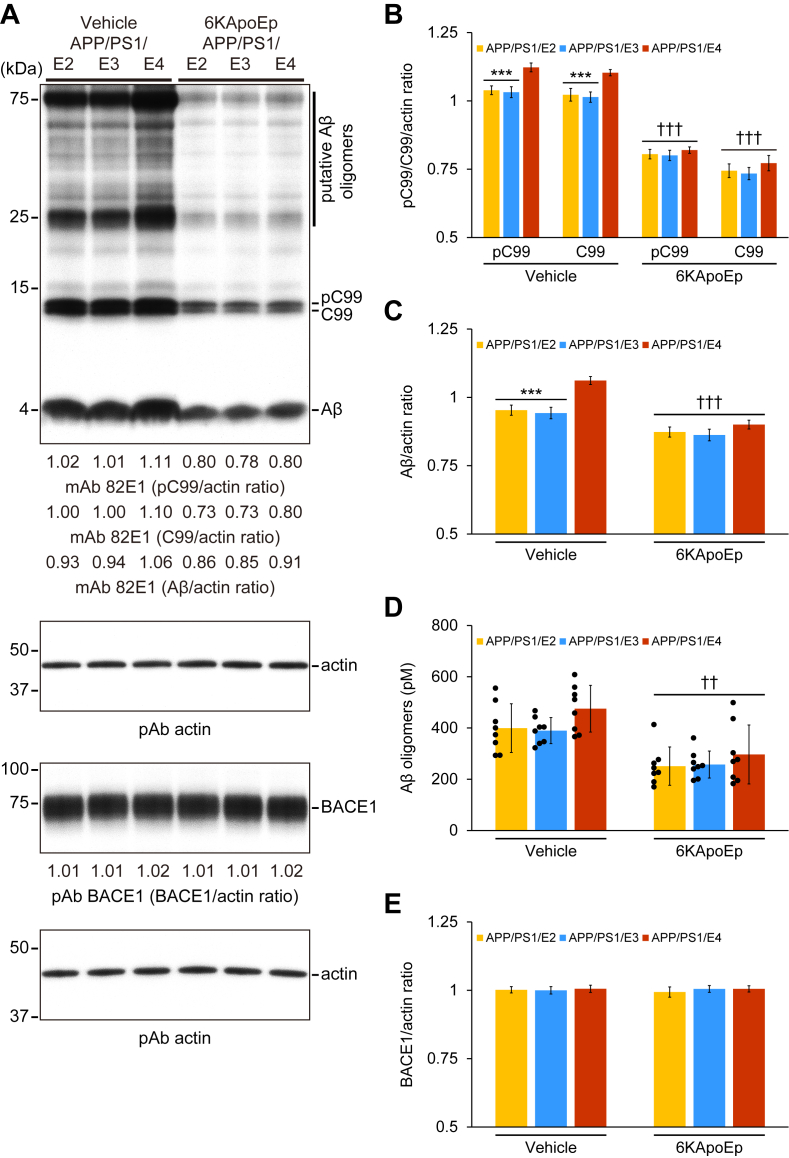
**6KApoEp treatment dampens amyloidogenic APP processing without altering BACE1 expression**. *A*, Western blot is shown using anti-N-terminal amyloid-β_1–17_ (*Aβ*) monoclonal antibody (*mAb 82E1*), which detects amyloidogenic APP cleavage fragments, including phospho-C99 (*pC99*) and nonphospho-C99 (*C99*) as well as Aβ monomer and oligomers. Western blot is also shown using anti-C-terminal BACE1 (β-secretase) polyclonal antibody (*pAb BACE1*). Actin is included as a loading control, and densitometry values are indicated *below* each lane. Equal amounts of total protein were loaded per lane. *B* and *C*, densitometry data are shown for ratios of pC99, C99, or Aβ to actin. *D*, abundance of Aβ oligomers in the detergent-soluble brain homogenate fraction (measured by sandwich ELISA) is shown. *E*, densitometry data are shown for ratios of BACE1 to actin. Data were obtained from APP/PS1/E2 mice that received vehicle (*APP/PS1/E2-V*) or 6KApoEp (*APP/PS1/E2-6KApoEp*), APP/PS1/E3 mice that received vehicle (*APP/PS1/E3-V*) or 6KApoEp (*APP/PS1/E3-6KApoEp*), and APP/PS1/E4 mice that received vehicle (*APP/PS1/E4-V*) or 6KApoEp (*APP/PS1/E4-6KApoEp*) for 3 months starting at 12 months of age. Western blotting data for (*B*, *C*, and *E*) included each mouse (*n* = 4 per group with two of each sex), and quantitative data were averaged. Sandwich ELISA data for (*D*) included each mouse (*n* = 8 per group with four of each sex), and measured data were averaged. Statistical comparisons for (*B*) are between groups for each protein. Statistical comparisons for (*C*–*E*) are between groups. ∗∗∗*p* ≤ 0.001 for APP/PS1/E2-V or APP/PS1/E3-V *versus* APP/PS1/E4-V mice; ^††^*p* < 0.01; ^†††^*p* ≤ 0.001 for each 6KApoEp- *versus* each vehicle-treated APP/PS1/E2/E3/E4 mice (Tables S16–S19). *V*, vehicle.

Subsequently, we investigated whether 6KApoEp therapy affects amyloidogenic APP processing. We probed brain homogenates for APP metabolites as follows: amyloidogenic phospho-β-CTF (pβ-CTF/pC99); β-CTF/C99; and monomeric/oligomeric Aβ species. In the densitometry analysis for pC99 and C99, two-way ANOVA denoted the main effects of genotype (*p* < 0.001) and treatment (*p* < 0.001) for pC99 and C99, and the interaction between them showed significance (*p* ≤ 0.001 for pC99; *p* < 0.05 for C99). Densitometry analysis confirmed that amyloidogenic APP cleavage to pC99 and C99 were significantly enhanced in APP/PS1/E4 mice *versus* APP/PS1/E2 or APP/PS1/E3 mice in the vehicle-treated mouse groups (Fig. 7, *A* and *B*; ∗∗∗*p* ≤ 0.001), and 6KApoEp therapy significantly inhibited pC99 and C99 band density (Fig. 7, *A* and *B*; ^†††^*p* < 0.001 for each 6KApoEp- *versus* each vehicle-treated APP/PS1/E2/E3/E4 mice), and significant differences were not displayed in *APOE* genotype in the 6KApoEp-treated mouse groups. Moreover, in the densitometry analysis for monomeric Aβ, two-way ANOVA demonstrated the main effects of genotype (*p* < 0.001) and treatment (*p* < 0.001), and the interaction between them showed significance (*p* < 0.001). The band density of the 4-kDa monomeric Aβ was significantly increased in vehicle-treated APP/PS1/E4 mice *versus* vehicle-treated APP/PS1/E2 or APP/PS1/E3 mice (Fig. 7, *A* and *C*; ∗∗∗*p* < 0.001). 6KApoEp therapy significantly reduced the band density of monomeric Aβ (Fig. 7, *A* and *C*; ^†††^*p* < 0.001 for each 6KApoEp- *versus* each vehicle-treated APP/PS1/E2/E3/E4 mice), and significant differences in *APOE* genotype did not disclose within 6KApoEp-treated APP/PS1/E2/E3/E4 mice. It appears that the ladder band density of Aβ species between 25 and 75 kDa (putative Aβ oligomers, Fig. 7*A*) augmented in vehicle-treated APP/PS1/E4 mice *versus* vehicle-treated APP/PS1/E2 or APP/PS1/E3 mice, and 6KApoEp therapy reduced expression of oligomeric Aβ species. This was further supported by sandwich ELISA for Aβ oligomers. In the biochemical analysis for Aβ oligomers, two-way ANOVA revealed the main effect of treatment (*p* < 0.001), and the interaction between genotype and treatment did not show significance, and 6KApoEp therapy significantly reduced abundance of Aβ oligomers (Fig. 7*D*; ^††^*p* < 0.01 for each 6KApoEp- *versus* each vehicle-treated APP/PS1/E2/E3/E4 mice). It is noteworthy that 6KApoEp-treated APP/PS1/E4 mice posted the greatest reductions across most measures of amyloidogenicity *versus* 6KApoEp-treated APP/PS1/E2 or APP/PS1/E3 mice.

These results suggest that 6KApoEp therapy modifies APP processing away from amyloidogenesis. To open the molecular keystones in the above findings, we examined BACE1 (β-secretase) proteins by Western blotting of brain homogenates from both treatment groups of APP/PS1/E2/E3/E4 mice. In the densitometry analysis for BACE1, two-way ANOVA did not reveal any significant effects of genotype and treatment, and nor any significant interaction between them. We found comparable expression of BACE1 protein between mouse strain and treatment groups (Fig. 7, *A* and *E*; *p* > 0.05). Therefore, 6KApoEp therapy did not affect amyloidogenic BACE1 expression but rather dampened APP amyloidogenesis due to reducing APP abundance at the plasma membrane.

### 6KApoEp therapy inhibits p44/42 MAPK phosphorylation and enhances p38 MAPK phosphorylation

**Figure 8:**
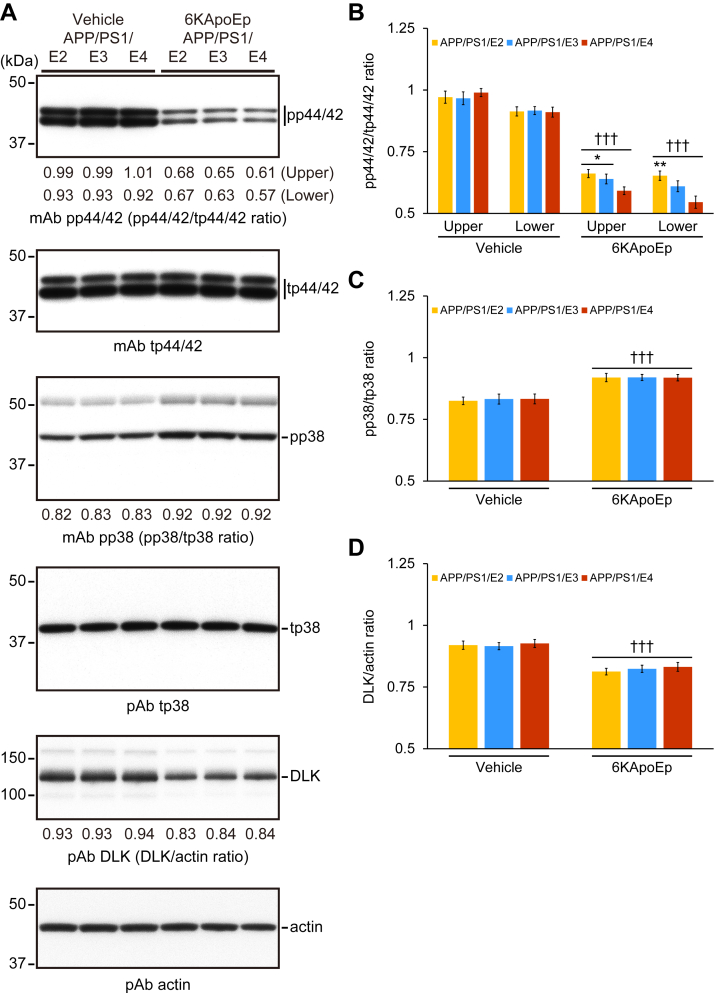
**6KApoEp therapy inhibits p44/42 MAPK phosphorylation and DLK expression**. *A*, Western blots are shown using anti-phospho-p44/42 MAPK monoclonal antibody (*mAb pp44/42*), anti-total p44/42 MAPK mAb (*mAb tp44/42*), anti-phospho-p38 MAPK mAb (*mAb pp38*), anti-total p38 MAPK polyclonal antibody (*pAb tp38*), or anti-DLK/MAP3K12 pAb (*pAb DLK*). Actin is included as an internal loading control, and densitometry values are shown *below* each lane. *B*–*D*, densitometry data are shown for ratios of pp44/42 to tp44/42, pp38 to tp38, and DLK to actin, respectively. Data were obtained from APP/PS1/E2 mice that received vehicle (*APP/PS1/E2-V*) or 6KApoEp (*APP/PS1/E2-6KApoEp*), APP/PS1/E3 mice that received vehicle (*APP/PS1/E3-V*) or 6KApoEp (*APP/PS1/E3-6KApoEp*), and APP/PS1/E4 mice that received vehicle (*APP/PS1/E4-V*) or 6KApoEp (*APP/PS1/E4-6KApoEp*) for 3 months starting at 12 months of age. Western blotting data for (*B*–*D*) included each mouse (*n* = 4 per group with two of each sex), and quantitative data were averaged. Statistical comparisons for (*B*) are between groups for each protein. Statistical comparisons for (*C* and *D*) are between groups. ∗*p* < 0.05; ∗∗*p* < 0.01 for APP/PS1/E2-6KApoEp or APP/PS1/E3-6KApoEp *versus* APP/PS1/E4-6KApoEp mice; ^†††^*p* < 0.001 for each 6KApoEp- *versus* each vehicle-treated APP/PS1/E2/E3/E4 mice (Tables S20–S22). *V*, vehicle.

ApoE-mediated *APP* transcription and Aβ production were found to be mediated by activation of the noncanonical p44/42 mitogen-activated protein kinase (MAPK) pathway (11). Previously, we investigated the effect of 6KApoEp on p44/42 MAPK and p38 MAPK phosphorylation and found that 6KApoEp inhibits apoE-induced p44/42 phosphorylation but activates p38 MAPK phosphorylation (12). Similarly, in the densitometry analysis for p44/42 and p38 MAPKs, two-way ANOVA denoted the main effects of genotype (*p* < 0.001) for the density of pp44/42 MAPK lower bands and treatment (*p* < 0.001) for the density of both pp44/42 MAPK upper and lower bands as well as pp38 MAPK bands. The interaction between them for the density of both pp44/42 MAPK upper and lower bands showed significances (*p* ≤ 0.001), while the interaction between them for the density of pp38 MAPK bands did not show significance. We found that 6KApoEp-treated APP/PS1/E2/E3/E4 mice displayed significantly reducing p44/42 MAPK phosphorylation while significantly enhancing p38 MAPK phosphorylation *versus* vehicle-treated APP/PS1/E2/E3/E4 mice, as determined by quantification of Western blotting (Fig. 8, *A*–*C*; ^†††^*p* ≤ 0.001 for each 6KApoEp- *versus* each vehicle-treated APP/PS1/E2/E3/E4 mice). Moreover, 6KApoEp therapy significantly decreased the density of both pp44/42 MAPK upper and lower bands in APP/PS1/E4 mice *versus* APP/PS1/E2 or APP/PS1/E3 mice (Fig. 8, *A* and *B*; ∗*p* < 0.05; ∗∗*p* < 0.01), and there were no significant differences within APP/PS1/E2/E3/E4 mice for the band density of pp38 MAPK.

Subsequently, we aimed to examine further the molecular mechanisms for reducing p44/42 MAPK phosphorylation by 6KApoEp therapy. As apoE binding to its receptors activates dual leucine-zipper kinase (DLK), a MAP kinase kinase kinase that then phosphorylates MKK7 and p44/42 MAPK (11), we investigated whether 6KApoEp therapy alters protein expression of DLK in brain homogenates from each group of APP/PS1/E2/E3/E4 mice by Western blotting. In the densitometry analysis for DLK, two-way ANOVA presented the main effect of treatment (*p* < 0.001), and the interaction between genotype and treatment did not show significance. We found that protein expression of DLK was significantly reduced in 6KApoEp-treated APP/PS1/E2/E3/E4 mouse brains, as determined by quantification of Western blotting (Fig. 8, *A* and *D*; ^†††^*p* < 0.001 for each 6KApoEp- *versus* each vehicle-treated APP/PS1/E2/E3/E4 mice).

Taken together, our findings suggest that 6KApoEp therapy reduces amyloidogenic APP processing by decreasing APP abundance at the plasma membrane and lessening *APP* transcription, potentially intermediated by the differential activation of MAPK pathways.

### 6KApoEp inhibits apoE-N-terminal APP interaction, and 6KApoEp is detected in brains from 6KApoEp-treated APP/PS1/E2/E3/E4 mice

In our previous study, we found that apoE interacts with N-terminal APP and that this interaction is inhibited by 6KApoEp (12). In the present study, we confirmed this physical association of apoE with APP *in vivo*. Brain homogenates from each vehicle-treated APP/PS1/E2/E3/E4 mice were immunoprecipitated with anti-N-terminal APP polyclonal antibody (pAb), followed by Western blotting with anti-C-terminal apoE pAb. Alternatively, the above brain homogenates were immunoprecipitated with anti-C-terminal apoE pAb, followed by Western blotting with anti-N-terminal APP pAb.

**Figure 9:**
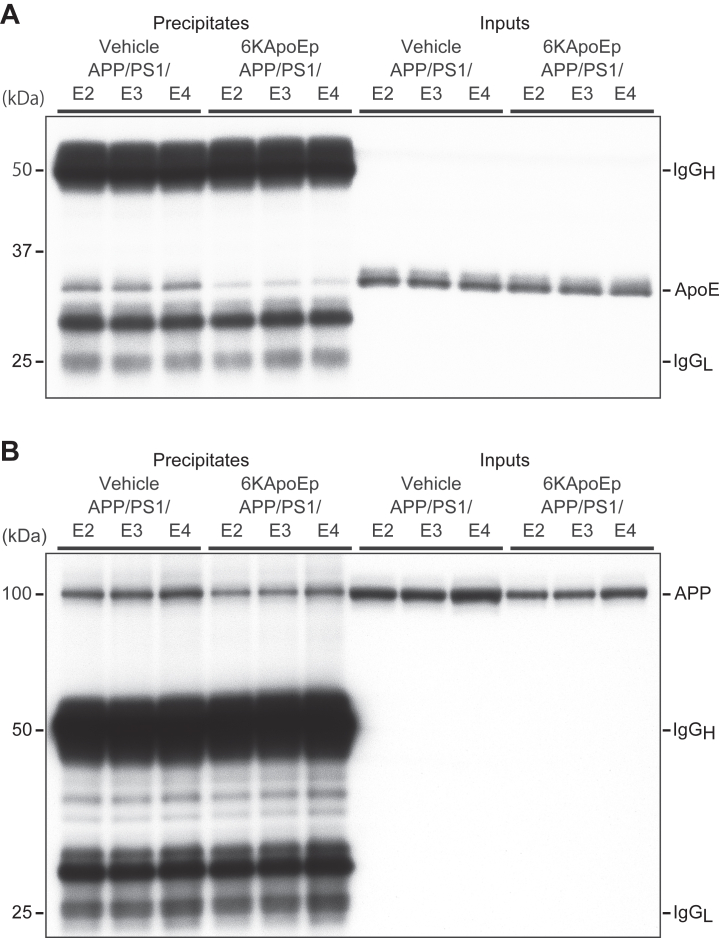
**6KApoEp inhibits apoE-N-terminal APP interaction**. *A* and *B*, brain homogenates from the vehicle-treated and 6KApoEp-treated APP/PS1/E2/E3/E4 mouse groups were immunoprecipitated with anti-N-terminal APP polyclonal antibody (*pAb*, *A*) or anti-C-terminal apoE pAb (*B*), and apoE (*A*) and APP (*holo*, *B*) were determined by Western blotting with anti-C-terminal apoE pAb or anti-N-terminal APP pAb. The *left six lanes* denote precipitates in each blot. The *right six lanes* denote inputs. IgG_H_, immunoglobulin heavy chain; IgG_L_, immunoglobulin light chain.

Our results demonstrated that apoE co-immunoprecipitates with APP (Fig. 9*A*) and that APP co-immunoprecipitates with apoE (Fig. 9*B*) in brain homogenates from each vehicle-treated APP/PS1/E2/E3/E4 mice, confirming that apoE physically associates with APP, and vice versa. Moreover, we found that 6KApoEp therapy hinders the above co-immunoprecipitation, as apoE and APP bands were greatly reduced (Fig. 9, *A* and *B*) in brain homogenates from each 6KApoEp-treated APP/PS1/E2/E3/E4 mice.

**Figure 10:**
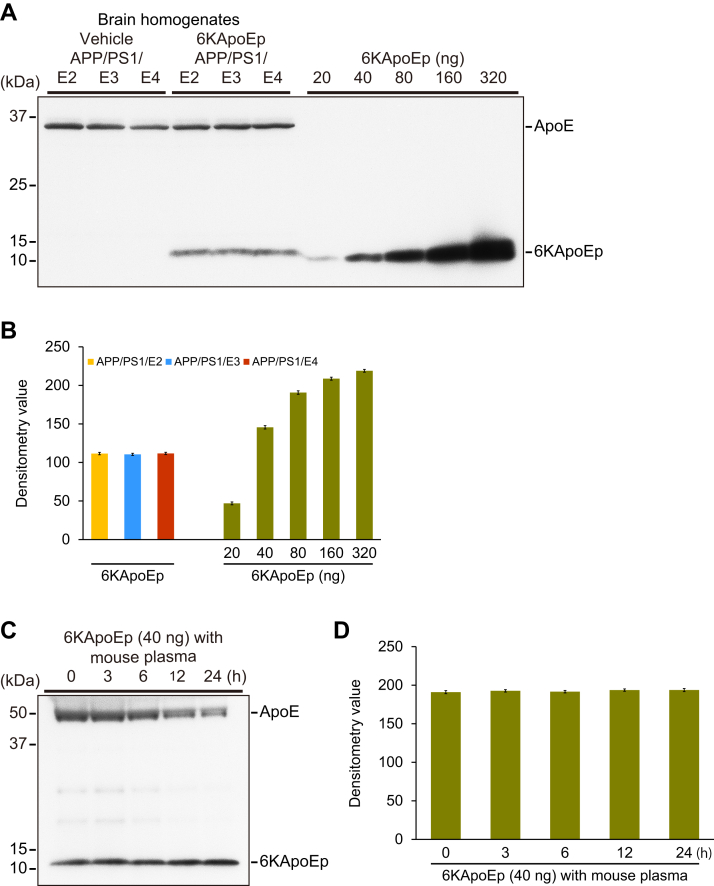
**6KApoEp is detected in brains from 6KApoEp-treated APP/PS1/E2/E3/E4 mice, and is stable in mouse plasma for 24 h at 37 °C.***A*, Western blots are shown using anti-low-density lipoprotein receptor (*LDLR*) binding domain (residues 133–152 of human apoE) polyclonal antibody (*pAb*) that is the matching epitope as 6KApoEp except for 6K. The *right five lanes* denote a series of 6KApoEp calibration peptides (*i.e.*, 20, 40, 80, 160, and 320 ng). Brain homogenates were obtained from APP/PS1/E2 mice that received vehicle (*APP/PS1/E2-V*) or 6KApoEp (*APP/PS1/E2-6KApoEp*), APP/PS1/E3 mice that received vehicle (*APP/PS1/E3-V*) or 6KApoEp (*APP/PS1/E3-6KApoEp*), and APP/PS1/E4 mice that received vehicle (*APP/PS1/E4-V*) or 6KApoEp (*APP/PS1/E4-6KApoEp*) for 3 months starting at 12 months of age. *B*, Western blotting data for the calibration graph were obtained from a series of 6KApoEp calibration peptides (*i.e.*, 20, 40, 80, 160, and 320 ng). Western blotting data for 3 bars were obtained from APP/PS1/E2 mice that received 6KApoEp (*APP/PS1/E2-6KApoEp*), APP/PS1/E3 mice that received 6KApoEp (*APP/PS1/E3-6KApoEp*), and APP/PS1/E4 mice that received 6KApoEp (*APP/PS1/E4-6KApoEp*) for 3 months starting at 12 months of age. Western blotting data for (*B*) included each mouse (*n* = 4 per group with two of each sex), and quantitative data were averaged. *C*, the stability of 6KApoEp (40 ng) in mouse plasma was examined with a time of incubation (*i.e.*, 0, 3, 6, 12, and 24 h) at 37 °C. *D*, the experiment was performed four times, and quantitative data were averaged (Table S23).

Finally, to examine whether 6KApoEp did enter the brain to interact with APP, we aimed to identify 6KApoEp in vehicle- and 6KApoEp-treated APP/PS1/E2/E3/E4 mouse brains by Western blotting with antibody against LDLR binding domain (residues 133–152) of human apoE that is the analogous epitope as 6KApoEp except for 6K. We detected 6KApoEp at the level of equivalent to between 20 and 40 ng/lane (10 μg protein) *versus* a series of 6KApoEp calibration peptides (*i.e.*, 20, 40, 80, 160, and 320 ng) in brain homogenates from APP/PS1/E2/E3/E4 mice with 3-month 6KApoEp therapy (Fig. 10, *A* and *B*). Moreover, we examined the stability of 6KApoEp in the mouse plasma at different incubation times (*i.e.*, 0, 3, 6, 12, and 24 h) at 37 °C by Western blotting using the same antibody. In the incubation of 6KApoEp with mouse plasma, 6KApoEp bands did not alter at any time of 37 °C incubation examined, whereas the intensity of the apoE band decreased over time upon incubation at 37 °C due to protein denaturation (Fig. 10, *C* and *D*).

Supporting information for raw data of Figure 1, *A*–*H* (Tables S24–S30); Figure 3, *D*–*F* (Tables S31–S33); Figure 6*B* (Table S34); Figure 7, *B*, *C* and *E* (Tables S35–S37); Figure 8, *B*–*D* (Tables S38–S40); Figure 10*B* (Table S41); and Figure 10*D* (Table S42) is shown.

## Discussion

Ideal drug development is to target a key molecular and/or specific interaction and to provide an agent that not only treats symptoms but essentially modifies disease with few side effects. We investigated the therapeutic potential of apoE-derived peptide, 6KApoEp that blocks the interaction of apoE with N-terminal APP for modifying AD-like pathology in APP/PS1 mice that express each apoE isoform of apoE2, apoE3, or apoE4.

Here, we report that intraperitoneal administration of 6KApoEp for 3 months completely reverses transgene-associated behavioral deficits (see Fig. 1). In parallel with learning and memory improvement, neuropathological analyses demonstrate that 6KApoEp therapy attenuates parenchymal and vascular amyloid pathology regardless of any apoE isoform carrier. Strikingly, the highest prophylactic effect for Aβ-lowering by 6KApoEp therapy is disclosed in APP/PS1/E4 mice *versus* the other two APP/PS1 mice harboring the apoE2 or apoE3 isoform (see Figs. 2 and 3).

In this regard, clinicopathological studies have demonstrated the link between cognitive decline and cerebral amyloid pathology in human cases with AD (31, 32). Consistent with our findings, the reversal of transgene-associated behavioral deficits through ameliorated cerebral amyloidosis has been allied in Alzheimer's mouse models (16, 17, 18, 19, 20, 21, 22, 33, 34, 35, 36, 37). In contrast, negative studies are that cognitive function and cerebral amyloidosis can be unfastened (33, 38). Indeed, elderly patients with AD having superior cognitive function reportedly have cerebral amyloid pathology comparable with prodromal or frank AD in autopsy brains (39, 40). One possible reason proposed for the discordancy between cognitive decline and cerebral amyloidosis in the literature is that soluble, oligomeric forms of Aβ are the candidate species for synapse toxicity (41, 42, 43, 44). This proposal is supported by findings in AD mouse models where behavioral impairment occurs in parallel with an increase of Aβ oligomers (35, 38). Noteworthy, we show that 6KApoEp therapy significantly decreases the abundance of Aβ oligomers in APP/PS1 mice carrying any apoE isoform (see Fig. 7), which correlates with improved cognitive function (see Fig. 1).

Synthesized APP in the endoplasmic reticulum is trafficked *via* the *trans*-Golgi network to the plasma membrane or further trafficked back into the cell by endocytosis (25, 26). In the sequential APP endoproteolysis, the amyloidogenic pathway generates C99 and soluble APP-β followed by Aβ production, whereas the nonamyloidogenic pathway generates C83 and soluble APP-α production (27, 28, 29, 30). We show that 6KApoEp therapy significantly decreased APP abundance in the plasma membrane-rich fraction of brain homogenates from APP/PS1 mice carrying any apoE isoform and that brain mRNA expression of *APP* slightly and significantly reduced in 6KApoEp-treated APP/PS1/E2/E3/E4 mice (see Fig. 6). Moreover, amyloidogenic pC99 and C99 in parallel with monomeric and oligomeric Aβ production are all decreased, providing evidence that the amyloidogenic APP proteolysis is dampened (see Fig. 7). Subsequently, we investigated whether 6KApoEp directly affects BACE1 that is responsible for the amyloidogenic APP processing and show that its protein expression did not alter (see Fig. 7).

An important question is how 6KApoEp acts on suppressing the amyloidogenic pathway. Previously, we and others have reported that apoE physically interacts with N-terminal APP and thereby effectively mediates the amyloidogenic APP processing into Aβ by enhancing intracellular APP endocytosis (10, 12). Interestingly, we have reported that 6KApoEp directly blocks this interaction and decreases APP trafficking to the plasma membrane and its subsequent endocytic APP processing (12). Hence, decreased abundance of APP itself at the plasma membrane shifts toward to reducing the amyloidogenic pathway.

A further question is how blockage of apoE and N-terminal APP interaction by 6KApoEp moves onto reducing APP trafficking to the plasma membrane. It has been reported that apoE binding to its receptors activates DLK, a MAP kinase kinase kinase and then phosphorylates MKK7 and p44/42 MAPK, which in turn enhances *APP* transcription and thereby increases Aβ production (11). In this regard, we have extended that apoE induces both p44/42 MAPK and p38 MAPK phosphorylation, which may affect APP endoproteolysis and subsequent Aβ production. Conversely, 6KApoEp therapy inhibits p44/42 MAPK phosphorylation but slightly induces p38 MAPK phosphorylation (12). Our current results are consistent with these previous findings. We further show that protein expression of DLK, an activator of p44/42 MAPK, is significantly reduced in 6KApoEp-treated APP/PS1/E2/E3/E4 mice. Thus, the reduction of DLK abundance by 6KApoEp therapy leads to suppressing p44/42 MAPK phosphorylation (see Fig. 8). Moreover, as p44/42 MAPK regulates sorting in early endosomes (45), reduced phosphorylation of p44/42 MAPK by 6KApoEp therapy may attenuate APP trafficking from the cell surface *via* endosomes. Yet, as MKK3, MKK4, and MKK6 are reported to phosphorylate p38 MAPK (46), these kinases may involve in the activation of p38 MAPK by 6KApoEp therapy.

Together, as apoE and 6KApoEp may differentially activate these MAPK pathways, thereby blocking apoE-N-terminal APP interaction, 6KApoEp may not halt some of the other apoE mediated physiological functions like lipid and cholesterol transportation *via* binding to LDLR on apoE.

In terms of bioavailability, it is important to note whether 6KApoEp does actually enter the brain. Based on a webserver B3Pred: a random-forest-based method for predicting and designing blood–brain barrier penetrating peptides (47), the designed therapeutic peptide, 6KApoEp (KKKKKK-LRVRLASHLRKLRKRLLRDA) was predicted as the blood–brain barrier penetrating peptide.

Moreover, in brain homogenates from APP/PS1/E2/E3/E4 mice with 6KApoEp therapy for 3 months, we detected 6KApoEp by Western blotting as the level of equivalent to between 20 and 40 ng/lane (10 μg protein) *versus* a series of 6KApoEp calibration peptides. Yet, according to our *ex vivo* data, the stability of 6KApoEp in mouse plasma was ensured for 24 h at 37 °C. (see Fig. 10). In the present study, we intraperitoneally administered 250 μg/kg/day of 6KApoEp to mice, which equates to 7.5 μg/day of 6KApoEp for the 30-g mouse. We extracted approximately 1800 μg of protein from a 60 mg brain piece for Western blotting, corresponding to 12,600 μg of protein for the whole brain (420 mg). Assuming the protein amount of the whole brain as the above, the level of 6KApoEp in the whole brain, estimated at 25.2 to 50.4 μg, is about 3.4 to 6.7 times the daily dose (7.5 μg). In this regard, as the equilibrium system (clearance system) toward brain–to–blood efflux exists in the brain (48), even if 6KApoEp reaches the brain parenchyma *via* the blood–brain barrier from the peripheral circulation, a certain amount of peptides may eliminate from the brain parenchyma with the equilibrium system without being retained in addition to the general loss of physiological metabolism and proteolysis. Therefore, it may be difficult to determine whether the estimated amount in the whole brain is underestimated, overestimated, or real. Future pharmacokinetic studies need to be done to determine the deposition, breakdown, and clearance of 6KApoEp over time to better explain its fate and level in the brain.

Consistent with the other reports from human AD cases (4) and AD mouse models (49, 50), we show that vehicle-treated APP/PS1/E4 mice demonstrate significant and isoform-specific deterioration of cerebral amyloid pathology *versus* vehicle-treated APP/PS1/E2 or APP/PS1/E3 mice (see Figure 2, Figure 3, Figure 4). Reported causes that increase the risk of AD with the carrying apoE4 isoform include an imbalance of Aβ equilibrium (balance of production and clearance) (51, 52), production of neurotoxic apoE peptide fragments (53), exacerbation of tau protein phosphorylation and aggregation (54), and neurodegeneration (55). We show that APP abundance at the plasma membrane significantly increases in vehicle-treated APP/PS1/E4 mice *versus* vehicle-treated APP/PS1/E2 or APP/PS1/E3 mice. As all three lines of vehicle-treated APP/PS1/E2, APP/PS1/E3, and APP/PS1/E4 mice disclosed comparable expression of BACE1 in their brain homogenates by Western blot analysis, aberrant APP transport or endocytosis would be the cause of deterioration in APP/PS1/E4 mice in addition to the previously reported causes.

Finally, both apoE3 and apoE4 have a much stronger binding affinity to their receptors *versus* apoE2 (13), and individuals carrying the lower-affinity apoE2 isoform are protected against AD and have much less brain pathology of cerebral amyloidosis as they age (56, 57). Thus, as the apoE-derived antagonistic peptide, 6KApoEp, might be expected to be particularly beneficial for patients with AD who are either apoE3 or apoE4 isoform carriers, we initially aimed at examining the apoE isoform-specific effect of 6KApoEp therapy. Contrary to our expectations, 6KApoEp did not have the same prophylactic effects in APP/PS1/E3 mice as in APP/PS1/E4 mice. Notably, 6KApoEp-treated APP/PS1/E4 mice demonstrated significant amelioration of cerebral amyloid pathology *versus* 6KApoEp-treated APP/PS1/E2 or APP/PS1/E3 mice. 6KApoEp may more specifically counteract the adverse effects of the apoE4 isoform by disturbing its binding to receptors.

Altogether, we report that administering the apoE-derived antagonistic peptide, 6KApoEp to the aged APP/PS1 mice of cerebral amyloidosis carrying each human apoE isoform of apoE2, apoE3, or apoE4 confers benefits on improving behavioral deficits, ameliorating cerebral amyloidosis, and reducing Aβ generation, which appears to have no detrimental effects of its own. In humans, several clinical trials of anti-amyloid therapies (*e.g.*, anti-Aβ antibodies or inhibitors of Aβ-generating secretases) for mild to moderate dementia of AD have failed due to unwanted side effects. As a shedding light, U.S. Food and Drug Administration has recently approved LEQEMBI (lecanemab-irmb) under the accelerated approval pathway as the disease-modifying drug for patients with mild cognitive impairment or mild dementia stage of AD. Nonetheless, drug development isn't over, and more effective disease-modifying drugs for moderate to advanced-stage disease must be developed. As 6KApoEp therapy ameliorates the adverse effect of apoE to promote Aβ production, it may serve as the basis for the development of human AD therapy. Thus, our fascinating approach may provide either as a stand-alone therapy or as part of a combination therapy and may be most suitable for cases with AD carrying the apoE4 isoform.

## Experimental procedures

### Ethics statement

All experiments were performed in accordance with the guidelines of the National Institutes of Health, and all animal studies were approved by the Saitama Medical University Institutional Animal Care and Use Committee. Animals were humanely cared for during all experiments, and all efforts were made to minimize suffering.

### Mice

APP/PS1 mice, male B6.Cg-Tg(APP_swe_, PSEN1dE9)85Dbo/Mmjax mice (bearing “Swedish” *APP*_K595N/M596L_ and *PS1* exon 9-deleted mutant human transgenes) (58) were obtained from The Jackson Laboratory (Bar Harbor, ME). Human apoE isoform knock-in mice expressing the human apoE2, apoE3, or apoE4 isoform in place of mouse apoE generated by homologous recombination (59, 60, 61) were gifted from Dr Shinobu C. Fujita (Mitsubishi Kagaku Institute of Life Sciences, Tokyo, Japan) and obtained from Riken BioResource Research Center (Tsukuba, Japan). Details on the production, genotyping, and genetic background of these mice are described in the source cited earlier.

To establish APP/PS1 mice expressing each human apoE isoform knock-in mice (APP/PS1/E2, APP/PS1/E3, or APP/PS1/E4 mice), male heterozygous APP/PS1 mice were bred to female homozygous E2, E3, or E4 mice. The resulting mice were then intercrossed to produce homozygous mice expressing each apoE isoform as well as WT offspring. At 12 months of age, these mice were used in this study, so all experimental mouse strains and WT littermates in this study are on the same genetic background.

The designed therapeutic peptide, 6KApoEp (KKKKKK-LRVRLASHLRKLRKRLLRDA), was synthesized by Hokkaido System Science Co, Ltd and kept with 1 mg per tube at −80 °C until use.

We randomly assigned APP/PS1/E2, APP/PS1/E3, or APP/PS1/E4 mice to two treatment groups (*n* = 8 per condition; four each sex): 6KApoEp (APP/PS1/E2-, APP/PS1/E3-, or APP/PS1/E4-6KApoEp) or vehicle (physiological saline; APP/PS1/E2-, APP/PS1/E3-, or APP/PS1/E4-V). Additionally, WT littermates received the same two treatments (*n* = 8 per group; four of each sex) as follows: WT-6KApoEp or vehicle (WT-V). After baseline behavioral assessment just prior to dosing (at 12 months of age), animals were intraperitoneally treated with 6KApoEp (250 μg/kg in 50 μl of physiological saline) or vehicle (50 μl of physiological saline) once daily for 3 months. The regent was freshly prepared daily prior to each treatment. Mice were housed in a specific pathogen-free barrier facility with a 12/12-h light/dark cycle and *ad libitum* access to food and water.

### Behavioral analyses

To assess episodic memory, mice were habituated in a cage for 4 h, and two objects of different shapes were concurrently provided for 10 min. The number of times that the animal explored the familiar object (defined as the number of instances where an animal directed its nose 2 cm or less from the object) was counted for the initial 5 min of exposure (training phase). To test memory retention on the following day, one of the familiar objects was replaced with a different-shaped novel object and explorations were recorded for 5 min (retention test). The recognition index, taken as a measurement of episodic memory, is reported as the frequency (%) of explorations of the novel *versus* familiar object (62).

To measure exploratory activity and spatial working memory, mice were individually placed in one arm of a radially symmetric Y-maze made of opaque gray acrylic (arms, 40 cm long and 4 cm wide; walls, 30 cm tall), and the sequence of arm entries and the total number of entries were counted over a period of 8 min, beginning when the animal first entered the central area. Percentage of alternation was defined as entries into sequentially different arms on consecutive occasions using the following formula: % alternation = number of alternations/(number of total arm entries − 2) × 100% (15).

To assess spatial reference learning and memory, the RAWM test was done over 2 days and consisted of triangular wedges in a circular pool (80 cm diameter) configured to form swim lanes that enclosed a central open space (63). Mice naïve to the task were placed in the pool and allowed to search for the platform for 60 s. Animals were dropped into a random start arm and allowed to swim until they located and climbed onto the platform (goal) over a period of 60 s. Latency to locate the platform and errors were recorded. Each mouse was given a total of 15 trials. On day 1, the goal alternated between visible and hidden, although on day 2, the goal was always hidden. All data were organized as individual blocks of three trials each. The goal arms remained in the same location for both days, whereas the start arm was randomly altered. All trials were done at the same time of day (±1 h), during the animals’ light phase. To avoid interference with behavioral testing, treatment was carried out 1 h after the conclusion of behavioral testing.

### Brain tissue preparation

At 15 months of age, anesthesia was induced and maintained with isoflurane (1.5%–2.0% and then 0.5%) after 12 h from the last peptide treatment. Mice were euthanized by transcardial perfusion with ice-cold physiological saline containing heparin (10 units/ml). Brains were isolated and quartered (sagittally at the level of the longitudinal fissure of the cerebrum, and then coronally at the level of the anterior commissure). Left anterior hemispheres were weighed and snap-frozen at −80 °C for Western blotting. Right anterior hemispheres were weighed and immersed in RNA stabilization solution (RNAlater, Applied Biosystems) and then snap-frozen at −80 °C for QPCR analysis. Left posterior hemispheres were immersed in 4% paraformaldehyde at 4 °C overnight and routinely processed in paraffin. Right posterior hemispheres including the hippocampus were weighed and snap-frozen at −80 °C for sandwich ELISA.

### Immunohistochemistry

Five coronal paraffin sections (per set) were cut with a 100-μm interval and 5-μm thickness spanning bregma −2.92 to −3.64 mm (64). One set of five sections was prepared for the analysis of β-amyloid plaques. Biotinylated anti-Aβ_17–24_ mAb (1:200 dilution, 4G8; Covance Research Products) was used as the primary antibody. Immunohistochemistry was performed using a Vectastain ABC *Elite* kit (Vector Laboratories) coupled with the diaminobenzidine reaction, except that the biotinylated secondary antibody step was omitted.

### Image analysis

Images were acquired and quantified using SimplePCI software (Hamamatsu Photonics). Images of five 5-μm sections through each anatomic ROI (*i.e.*, RSC, EC, and H) were captured based on anatomical criteria (64), and we set a threshold optical density that discriminated staining from the background. Selection bias was controlled for by analyzing each ROI in its entirety. For Aβ burden analysis, data are reported as the percentage of positive pixels captured divided by the full area captured. Anti-Aβ_17–24_ mAb, which recognizes amino acids 18 to 22 (VFFAE), was used for conventional Aβ burden analysis.

For β-amyloid plaque morphometric analysis, diameters (maximum lengths) of β-amyloid plaques were blindly measured and assigned to one of three mutually exclusive plaque size categories (<25, between 25 and 50, or >50 μm). For quantitative analysis of CAA, numbers of Aβ antibody-positive cerebral vessels were blindly counted in each ROI.

### ELISA

Brain Aβ_1–40_ and Aβ_1–42_ species were detected by a three-step extraction protocol with modifications (65). Brains were homogenized using TissueLyser LT (Qiagen) in Tris-buffered saline (TBS: 25 mM Tris-HCl, pH 7.4, 150 mM NaCl) containing protease inhibitor mixture (Sigma-Aldrich) and phosphatase inhibitor tablet (Roche), centrifuged at 18,800*g* for 60 min at 4 °C, and supernatants were collected (representing the TBS-soluble fraction). The resulting pellets were treated with TNE buffer (10 mM Tris-HCl, 1% Nonidet P-40, 1 mM EDTA, and 150 mM NaCl) with protease and phosphatase inhibitors, and homogenized using TissueLyser LT. Homogenates were then centrifuged at 18,800*g* for 30 min at 4 °C, and supernatants were collected (representing the detergent-soluble fraction). The remaining pellets were treated with 5 M guanidine-HCl and dissolved by occasional mixing on ice for 30 min and centrifuged at 18,800*g* for 30 min at 4 °C. Supernatants were then collected; this is taken as the guanidine-HCl-soluble fraction. Aβ_1–40_ and Aβ_1–42_ species were separately quantified in each sample in duplicate by sandwich ELISA (IBL) (66). Aβ oligomers were quantified in the TBS-soluble fraction in duplicate by human Aβ oligomers (82E1-specific) assay (IBL) (67). All samples fell within the linear range of the standard curve.

### Western blotting

Brain homogenates were lysed using TissueLyser LT in TBS containing protease inhibitor mixture (Sigma-Aldrich) and phosphatase inhibitor tablet (Roche) followed by TNE buffer. Homogenates were then centrifuged at 18,800*g* for 30 min at 4 °C, supernatants were collected, and aliquots corresponding to 10 μg of total protein were electrophoretically separated using Tris-glycine gels. Electrophoresed proteins were transferred to polyvinylidene difluoride membranes (Bio-Rad) that were blocked at ambient temperature for 1 h. Membranes were then hybridized at ambient temperature for 1 h with primary antibodies as follows: anti-N-terminal APP pAb (1:2000 dilution, IBL); anti-sodium-potassium ATPase mAb (1:20,000 dilution, EP1845Y; abcam), anti-heat-shock protein 90 mAb (1:1000 dilution, C45G5; Cell Signaling Technology), anti-N-terminal Aβ_1–16_ mAb (1:500 dilution, 82E1; IBL); anti-C-terminal-BACE1 pAb (1:400 dilution; IBL); anti-phospho-p44/42 MAPK mAb (1:2000 dilution, D13.14.4E; Cell Signaling Technology); anti-p44/42 MAPK mAb (1:3000 dilution, 137F5; Cell Signaling Technology); anti-phospho-p38 MAPK (Thr180/Tyr182) mAb (1:1000 dilution, D3F9; Cell Signaling Technology); anti-p38 MAPK pAb (1:1500 dilution, Cell Signaling Technology); anti-C-terminal apoE pAb, (1:1000 dilution, A299; IBL); anti-human apoE LDLR binding domain pAb (1:1000 dilution; IBL), anti-DLK/MAP3K12 pAb (1:1500 dilution, GeneTex, Inc), or anti-β-actin mAb (1:5000 dilution, 13E5; Cell Signaling Technology; as a loading control). Membranes were then rinsed and incubated at ambient temperature for 1 h with appropriate horseradish peroxidase-conjugated secondary antibodies. After additional rinsing, membranes were incubated at ambient temperature for 5 min with enhanced chemiluminescence substrate (SuperSignal West Dura Extended Duration Substrate, Thermo Fisher Scientific), exposed to film, and developed. Western blots were done for each brain (*n* = 4, two mice of each sex per group), and quantitative data were averaged. The intensity of each band by Western blotting was quantified using SimplePCI software (Hamamatsu Photonics).

### Co-immunoprecipitation

Brain homogenates were lysed using TissueLyser LT in TBS containing protease inhibitor mixture (Sigma-Aldrich) and phosphatase inhibitor tablet (Roche) followed by TNE buffer. Homogenates were then centrifuged at 18,800*g* for 30 min at 4 °C, supernatants were collected.

Co-immunoprecipitation was performed according to the manufacturer’s instructions.

Aliquots corresponding to 500 μg of total protein were immunoprecipitated with anti-N-terminal pAb (IBL) or anti-C-terminal apoE pAb, (A299; IBL) and Dynabeads Protein G (Thermo Fisher Scientific) for 1 h with gentle rocking at room temperature, followed by three washes with TNE buffer and analysis by Western blotting with anti-C-terminal apoE pAb, (1:1000 dilution, A299; IBL) or anti-N-terminal APP pAb (1:2000 dilution, IBL).

### Quantitative real-time PCR

We quantified *App* and *β-actin* mRNA levels by QPCR analysis. Total RNA was extracted using the RNeasy mini kit (Qiagen), and first-strand cDNA synthesis was carried out using the QuantiTect reverse transcription kit (Qiagen) in accordance with the manufacturer’s instructions. We diluted cDNA 1:1 in H_2_O and carried out QPCR for all genes of interest using cDNA-specific TaqMan primer/probe sets (TaqMan Gene Expression Assays, Applied Biosystems) on an ABI 7500 Fast real-time PCR instrument (Applied Biosystems). Each 20-μl reaction mixture contained 2 μl of cDNA with 1 μl of TaqMan Gene Expression Assay reagent, 10 μl of TaqMan Fast Universal PCR Master Mix (Applied Biosystems), and 7 μl of H_2_O. Thermocycler conditions consisted of 95 °C for 15 s, followed by 40 cycles of 95 °C for 1 s and 60 °C for 20 s. Mouse TaqMan probe/primer sets were as follows: *App* (number Mm01344172_m1); and *β-actin* (number Mm00607939_s1; used as an internal reference control) (Applied Biosystems). Samples that were not subjected to reverse transcription were run in parallel as negative controls to rule out genomic DNA contamination, and a “no template control” was also included for each primer set. The cycle threshold number (*C*_T_) method (68) was used to determine relative amounts of initial target cDNA in each sample. Results were expressed relative to vehicle-treated APP/PS1/E2 mice.

### Statistical analysis

Data are presented as means with associated standard deviations. A hierarchical analysis strategy was used in which we first conducted two-way ANOVA to assess the significance for the main effects of genotype and treatment, and the interaction between them. If the model was significant, post hoc testing was done with Tukey’s HSD or Dunnett’s T3 methods, where appropriateness was determined based on Levene’s test for equality of the variance. If the interaction was not significant, but the main effects of genotype and/or treatment were significant, post hoc testing for genotype and/or treatment was done with Tukey’s HSD or Dunnett’s T3 methods (depending on Levene’s test for equality of the variance) as well as *t* test for two samples. In instances of RAWM behavioral data, ANOVA (two-way and repeated measures) was used, followed by post hoc comparison of the means using Tukey’s HSD or Dunnett’s T3 methods (depending on Levene’s test for equality of the variance). In instances of 6KApoEp stability data, one-way ANOVA was used. The α levels were set at 0.05 for all analyses. All analyses were performed using the Statistical Package for the Social Sciences, release 23.0 (IBM SPSS).

## Data availability

All data described in this manuscript are contained within the manuscript.

## Supporting information

This article contains supporting information.

## Conflict of interest

The authors except for Darrell Sawmiller declare that they have no conflicts of interest with the contents of this article. Darrell Sawmiller has a patent application for 6KApoEp submitted.

## References

[1] Selkoe D.J. (2001). Alzheimer’s disease: genes, proteins, and therapy. Physiol. Rev..

[2] Mahley R.W. (1988). Apolipoprotein E: cholesterol transport protein with expanding role in cell biology. Science.

[3] Poirier J. (1994). Apolipoprotein E in animal models of CNS injury and in Alzheimer’s disease. Trends Neurosci..

[4] Corder E.H., Saunders A.M., Strittmatter W.J., Schmechel D.E., Gaskell P.C., Small G.W. (1993). Gene dose of apolipoprotein E type 4 allele and the risk of Alzheimer's disease in late onset families. Science.

[5] Corder E.H., Saunders A.M., Risch N.J., Strittmatter W.J., Schmechel D.E., Gaskell P.C. (1994). Protective effect of apolipoprotein E type 2 allele for late onset Alzheimer disease. Nat. Genet..

[6] Namba Y., Tomonaga M., Kawasaki H., Otomo E., Ikeda K. (1991). Apolipoprotein E immunoreactivity in cerebral amyloid deposits and neurofibrillary tangles in Alzheimer's disease and kuru plaque amyloid in Creutzfeldt-Jakob disease. Brain Res..

[7] Wisniewski T., Frangione B. (1992). Apolipoprotein E: a pathological chaperone protein in patients with cerebral and systemic amyloid. Neurosci. Lett..

[8] Strittmatter W.J., Saunders A.M., Schmechel D., Pericak-Vance M., Enghild J., Salvesen G.S. (1993). Apolipoprotein E: high-avidity binding to β-amyloid and increased frequency of type 4 allele in late-onset familial Alzheimer disease. Proc. Natl. Acad. Sci. U. S. A..

[9] LaDu M.J., Falduto M.T., Manelli A.M., Reardon C.A., Getz G.S., Frail D.E. (1994). Isoform-specific binding of apolipoprotein E to β-amyloid. J. Biol. Chem..

[10] Hass S., Fresser F., Köchl S., Beyreuther K., Utermann G., Baier G. (1998). Physical interaction of ApoE with amyloid precursor protein independent of the amyloid Aβ region *in vitro*. J. Biol. Chem..

[11] Huang Y.-W.A., Zhou B., Wernig M., Südhof T.C. (2017). ApoE2, ApoE3, and ApoE4 differentially stimulate APP transcription and Aβ secretion. Cell.

[12] Sawmiller D., Habib A., Hou H., Mori T., Fan A., Tian J. (2019). A novel apolipoprotein E antagonist functionally blocks apolipoprotein E interaction with N-terminal amyloid precursor protein, reduces β-amyloid-associated pathology, and improves cognition. Biol. Psychiatry.

[13] Bu G. (2009). Apolipoprotein E and its receptors in Alzheimer’s disease: pathways, pathogenesis and therapy. Nat. Rev. Neurosci..

[14] King D.L., Arendash G.W., Crawford F., Sterk T., Menendez J., Mullan M.J. (1999). Progressive and gender-dependent cognitive impairment in the APP_SW_ transgenic mouse model for Alzheimer's disease. Behav. Brain Res..

[15] Arendash G.W., King D.L., Gordon M.N., Morgan D., Hatcher J.M., Hope C.E. (2001). Progressive, age-related behavioral impairments in transgenic mice carrying both mutant amyloid precursor protein and presenilin-1 transgenes. Brain Res..

[16] Town T., Laouar Y., Pittenger C., Mori T., Szekely C.A., Tan J. (2008). Blocking TGF-β-Smad2/3 innate immune signaling mitigates Alzheimer-like pathology. Nat. Med..

[17] Mori T., Rezai-Zadeh K., Koyama N., Arendash G.W., Yamaguchi H., Kakuda N. (2012). Tannic acid is a natural β-secretase inhibitor that prevents cognitive impairment and mitigates Alzheimer-like pathology in transgenic mice. J. Biol. Chem..

[18] Mori T., Koyama N., Guillot-Sestier M.V., Tan J., Town T. (2013). Ferulic acid is a nutraceutical β-secretase modulator that improves behavioral impairment and Alzheimer-like pathology in transgenic mice. PLoS One.

[19] Mori T., Koyama N., Segawa T., Maeda M., Maruyama N., Kinoshita N. (2014). Methylene blue modulates β-secretase, reverses cerebral amyloidosis, and improves cognition in transgenic mice. J. Biol. Chem..

[20] Mori T., Koyama N., Tan J., Segawa T., Maeda M., Town T. (2017). Combination therapy with octyl gallate and ferulic acid improves cognition and neurodegeneration in a transgenic mouse model of Alzheimer disease. J. Biol. Chem..

[21] Mori T., Koyama N., Tan J., Segawa T., Maeda M., Town T. (2019). Combined treatment with the phenolics (−)-epigallocatechin-3-gallate and ferulic acid improves cognition and reduces Alzheimer-like pathology in mice. J. Biol. Chem..

[22] Mori T., Koyama N., Yokoo T., Segawa T., Maeda M., Sawmiller D. (2020). Gallic acid is a dual α/β-secretase modulator that reverses cognitive impairment and remediates pathology in Alzheimer mice. J. Biol. Chem..

[23] Kim K.S., Han P.L. (2006). Optimization of chronic stress paradigms using anxiety- and depression-like behavioral parameters. J. Neurosci. Res..

[24] Ellis R.J., Olichney J.M., Thal L.J., Mirra S.S., Morris J.C., Beekly D. (1996). Cerebral amyloid angiopathy in the brains of patients with Alzheimer’s disease: the CERAD experience, Part XV. Neurology.

[25] Haass C., Selkoe D.J. (1993). Cellular processing of β-amyloid precursor protein and the genesis of amyloid β-peptide. Cell.

[26] Choy R.W.-Y., Cheng Z., Schekman R. (2012). Amyloid precursor protein (APP) traffics from the cell surface via endosomes for amyloid β (Aβ) production in the *trans*-Golgi network. Proc. Natl. Acad. Sci. U. S. A..

[27] De Strooper B., Saftig P., Craessaerts K., Vanderstichele H., Guhde G., Annaert W. (1998). Deficiency of presenilin-1 inhibits the normal cleavage of amyloid precursor protein. Nature.

[28] Sinha S., Lieberburg I. (1999). Cellular mechanisms of β-amyloid production and secretion. Proc. Natl. Acad. Sci. U. S. A..

[29] Vassar R., Bennett B.D., Babu-Khan S., Kahn S., Mendiaz E.A., Denis P. (1999). β-Secretase cleavage of Alzheimer’s amyloid precursor protein by the transmembrane aspartic protease BACE. Science.

[30] Kimberly W.T., LaVoie M.J., Ostaszewski B.L., Ye W., Wolfe M.S., Selkoe D.J. (2003). γ-Secretase is a membrane protein complex comprised of presenilin, nicastrin, Aph-1, and Pen-2. Proc. Natl. Acad. Sci. U. S. A..

[31] Sabbagh M.N., Cooper K., DeLange J., Stoehr J.D., Thind K., Lahti T. (2010). Functional, global and cognitive decline correlates to accumulation of Alzheimer's pathology in MCI and AD. Curr. Alzheimer Res..

[32] Robinson J.L., Geser F., Corrada M.M., Berlau D.J., Arnold S.E., Lee V.M. (2011). Neocortical and hippocampal amyloid-β and tau measures associate with dementia in the oldest-old. Brain.

[33] Holcomb L.A., Gordon M.N., Jantzen P., Hsiao K., Duff K., Morgan D. (1999). Behavioral changes in transgenic mice expressing both amyloid precursor protein and presenilin-1 mutations: lack of association with amyloid deposits. Behav. Genet..

[34] Schenk D., Barbour R., Dunn W., Gordon G., Grajeda H., Guido T. (1999). Immunization with amyloid-β attenuates Alzheimer-disease-like pathology in the PDAPP mouse. Nature.

[35] Kotilinek L.A., Bacskai B., Westerman M., Kawarabayashi T., Younkin L., Hyman B.T. (2002). Reversible memory loss in a mouse transgenic model of Alzheimer’s disease. J. Neurosci..

[36] Hartman R.E., Izumi Y., Bales K.R., Paul S.M., Wozniak D.F., Holtzman D.M. (2005). Treatment with an amyloid-β antibody ameliorates plaque load, learning deficits, and hippocampal long-term potentiation in a mouse model of Alzheimer's disease. J. Neurosci..

[37] Mouri A., Noda Y., Hara H., Mizoguchi H., Tabira T., Nabeshima T. (2007). Oral vaccination with a viral vector containing Aβ cDNA attenuates age-related Aβ accumulation and memory deficits without causing inflammation in a mouse Alzheimer model. FASEB J..

[38] Westerman M.A., Cooper-Blacketer D., Mariash A., Kotilinek L., Kawarabayashi T., Younkin L.H. (2002). The relationship between Aβ and memory in the Tg2576 mouse model of Alzheimer’s disease. J. Neurosci..

[39] Price J.L., McKeel D.W., Buckles V.D., Roe C.M., Xiong C., Grundman M. (2009). Neuropathology of nondemented aging: presumptive evidence for preclinical Alzheimer disease. Neurobiol. Aging.

[40] Mufson E.J., Malek-Ahmadi M., Perez S.E., Chen K. (2016). Braak staging, plaque pathology, and APOE status in elderly persons without cognitive impairment. Neurobiol. Aging.

[41] Walsh D.M., Klyubin I., Fadeeva J.V., Cullen W.K., Anwyl R., Wolfe M.S. (2002). Naturally secreted oligomers of amyloid β protein potently inhibit hippocampal long-term potentiation *in vivo*. Nature.

[42] Cleary J.P., Walsh D.M., Hofmeister J.J., Shankar G.M., Kuskowski M.A., Selkoe D.J. (2005). Natural oligomers of the amyloid-β protein specifically disrupt cognitive function. Nat. Neurosci..

[43] Haass C., Selkoe D.J. (2007). Soluble protein oligomers in neurodegeneration: lessons from the Alzheimer’s amyloid β-peptide. Nat. Rev. Mol. Cell Biol..

[44] Shankar G.M., Li S., Mehta T.H., Garcia-Munoz A., Shepardson N.E., Smith I. (2008). Amyloid-β protein dimers isolated directly from Alzheimer’s brains impair synaptic plasticity and memory. Nat. Med..

[45] Kostenko O., Tsacoumangos A., Crooks D., Kil S.J., Carlin C. (2006). Gab1 signaling is regulated by EGF receptor sorting in early endosomes. Oncogene.

[46] Canovas B., Nebreda A.R. (2021). Diversity and versatility of p38 kinase signalling in health and disease. Nat. Rev. Mol. Cell Biol..

[47] Kumar V., Patiyal S., Dhall A., Sharma N., Raghava G.P.S. (2021). B3Pred: a random-forest-based method for predicting and designing blood-brain barrier penetrating peptides. Pharmaceutics.

[48] DeMattos R.B., Bales K.R., Cummins D.J., Paul S.M., Holtzman D.M. (2002). Brain to plasma amyloid-β efflux: a measure of brain amyloid burden in a mouse model of Alzheimer’s disease. Science.

[49] Bales K.R., Liu F., Wu S., Lin S., Koger D., DeLong C. (2009). Human *APOE* isoform-dependent effects on brain β-amyloid levels in PDAPP transgenic mice. J. Neurosci..

[50] Youmans K.L., Tai L.M., Nwabuisi-Heath E., Jungbauer L., Kanekiyo T., Gan M. (2012). *APOE*4-specific changes in Aβ accumulation in a new transgenic mouse model of Alzheimer disease. J. Biol. Chem..

[51] Ye S., Huang Y., Müllendorff K., Dong L., Giedt G., Meng E.C. (2005). Apolipoprotein (apo) E4 enhances amyloid β peptide production in cultured neuronal cells: apoE structure as a potential therapeutic target. Proc. Natl. Acad. Sci. U. S. A..

[52] Castellano J.M., Kim J., Stewart F.R., Jiang H., DeMattos R.B., Patterson B.W. (2011). Human apoE isoforms differentially regulate brain amyloid-β peptide clearance. Sci. Transl. Med..

[53] Harris F.M., Brecht W.J., Xu Q., Tesseur I., Kekonius L., Wyss-Coray T. (2003). Carboxyl-terminal-truncated apolipoprotein E4 causes Alzheimer's disease-like neurodegeneration and behavioral deficits in transgenic mice. Proc. Natl. Acad. Sci. U. S. A..

[54] Brecht W.J., Harris F.M., Chang S., Tesseur I., Yu G.-Q., Xu Q. (2004). Neuron-specific apolipoprotein E4 proteolysis is associated with increased tau phosphorylation in brains of transgenic mice. J. Neurosci..

[55] Shi Y., Yamada K., Liddelow S.A., Smith S.T., Zhao L., Luo W. (2017). ApoE4 markedly exacerbates tau-mediated neurodegeneration in a mouse model of tauopathy. Nature.

[56] Benjamin R., Leake A., McArthur F.K., Ince P.G., Candy J.M., Edwardson J.A. (1994). Protective effect of apoE ϵ2 in Alzheimer's disease. Lancet.

[57] Suri S., Heise V., Trachtenberg A.J., Mackay C.E. (2013). The forgotten *APOE* allele: a review of the evidence and suggested mechanisms for the protective effect of *APOE* ϵ2. Neurosci. Biobehav. Rev..

[58] Jankowsky J.L., Slunt H.H., Gonzales V., Jenkins N.A., Copeland N.G., Borchelt D.R. (2004). APP processing and amyloid deposition in mice haplo-insufficient for presenilin 1. Neurobiol. Aging.

[59] Hamanaka H., Katoh-Fukui Y., Suzuki K., Kobayashi M., Suzuki R., Motegi Y. (2000). Altered cholesterol metabolism in human apolipoprotein E4 knock-in mice. Hum. Mol. Genet..

[60] Mori T., Kobayashi M., Town T., Fujita S.C., Asano T. (2003). Increased vulnerability to focal ischemic brain injury in human apolipoprotein E4 knock-in mice. J. Neuropathol. Exp. Neurol..

[61] Mori T., Town T., Kobayashi M., Tan J., Fujita S.C., Asano T. (2004). Augmented delayed infarct expansion and reactive astrocytosis after permanent focal ischemia in apolipoprotein E4 knock-in mice. J. Cereb. Blood Flow Metab..

[62] De Rosa R., Garcia A.A., Braschi C., Capsoni S., Maffei L., Berardi N. (2005). Intranasal administration of nerve growth factor (NGF) rescues recognition memory deficits in AD11 anti-NGF transgenic mice. Proc. Natl. Acad. Sci. U. S. A..

[63] Alamed J., Wilcock D.M., Diamond D.M., Gordon M.N., Morgan D. (2006). Two-day radial-arm water maze learning and memory task; robust resolution of amyloid-related memory deficits in transgenic mice. Nat. Protoc..

[64] Franklin K.B.J., Paxinos G. (2001).

[65] Kawarabayashi T., Younkin L.H., Saido T.C., Shoji M., Ashe K.H., Younkin S.G. (2001). Age-dependent changes in brain, CSF, and plasma amyloid (β) protein in the Tg2576 transgenic mouse model of Alzheimer’s disease. J. Neurosci..

[66] Horikoshi Y., Sakaguchi G., Becker A.G., Gray A.J., Duff K., Aisen P.S. (2004). Development of Aβ terminal end-specific antibodies and sensitive ELISA for Aβ variant. Biochem. Biophys. Res. Commun..

[67] Xia W., Yang T., Shankar G., Smith I.M., Shen Y., Walsh D.M. (2009). A specific enzyme-linked immunosorbent assay for measuring β-amyloid protein oligomers in human plasma and brain tissue of patients with Alzheimer disease. Arch. Neurol..

[68] Monney L., Sabatos C.A., Gaglia J.L., Ryu A., Waldner H., Chernova T. (2002). Th1-specific cell surface protein Tim-3 regulates macrophage activation and severity of an autoimmune disease. Nature.

