# Supplementary material for: Targeting apolipoprotein E and N-terminal amyloid β-protein precursor interaction improves cognition and reduces amyloid pathology in Alzheimer’s mice
Source: J Biol Chem. 2023 May 19;299(7):104846. doi: 10.1016/j.jbc.2023.104846 (PMC10331488; doi:10.1016/j.jbc.2023.104846)
Supplement: Supporting Tables [file mmc1.pdf]

Targeting apolipoprotein E and N-terminal amyloid β-protein precursor interaction improves cognition and reduces amyloid pathology in Alzheimer mice

Darrell Sawmiller, Naoki Koyama, Masakazu Fujiwara,
Tatsuya Segawa, Masahiro Maeda, and Takashi Mori

Supporting information

Table S1 (Supporting information of Fig. 1, *A* and *B*)

Object recognition test

Training

Two-way ANOVA

Factor	*p* value
Genotype	0.978
Treatment	0.921
Genotype * Treatment	0.942

Retention test

Two-way ANOVA

Factor	*p* value
Genotype	0.012
Treatment	0.000
Genotype * Treatment	0.094

**Group comparison**	**Factor**	***p* value**	***post hoc* test**
WT-V *versus* APP/PS1/E2-V	Genotype	0.009	Tukey's HSD method
WT-V *versus* APP/PS1/E3-V	Genotype	0.005	Tukey's HSD method
WT-V *versus* APP/PS1/E4-V	Genotype	0.012	Tukey's HSD method
WT-6KApoEp *versus* APP/PS1/E2-6KApoEp	Genotype	0.913	Tukey's HSD method
WT-6KApoEp *versus* APP/PS1/E3-6KApoEp	Genotype	0.942	Tukey's HSD method
WT-6KApoEp *versus* APP/PS1/E4-6KApoEp	Genotype	0.994	Tukey's HSD method
WT-V *versus* WT-6KApoEp	Treatment	0.551	*t* -test for two samples
APP/PS1/E2-V *versus* APP/PS1/E2-6KApoEp	Treatment	0.004	*t* -test for two samples
APP/PS1/E3-V *versus* APP/PS1/E3-6KApoEp	Treatment	0.002	*t* -test for two samples
APP/PS1/E4-V *versus* APP/PS1/E4-6KApoEp	Treatment	0.003	*t* -test for two samples

Table S2 (Supporting information of Fig. 1*C*)

Y-maze test

Number of arms entered

**Two-way ANOVA**	**Factor**	***p* value**
	Genotype	0.031
	Treatment	0.000
	Genotype * Treatment	0.028

**Group comparison**	**Factor**	***p* value**	***post hoc* test**
WT-V *versus* APP/PS1/E2-V	Genotype	0.008	Tukey's HSD method
WT-V *versus* APP/PS1/E3-V	Genotype	0.023	Tukey's HSD method
WT-V *versus* APP/PS1/E4-V	Genotype	0.026	Tukey's HSD method
APP/PS1/E2-V *versus* APP/PS1/E3-V	Genotype	1.000	Tukey's HSD method
APP/PS1/E2-V *versus* APP/PS1/E4-V	Genotype	1.000	Tukey's HSD method
WT-6KApoEp *versus* APP/PS1/E2-6KApoEp	Genotype	1.000	Tukey's HSD method
WT-6KApoEp *versus* APP/PS1/E3-6KApoEp	Genotype	1.000	Tukey's HSD method
WT-6KApoEp *versus* APP/PS1/E4-6KApoEp	Genotype	1.000	Tukey's HSD method
APP/PS1/E2-V *versus* APP/PS1/E2-6KApoEp	Treatment	0.011	Tukey's HSD method
APP/PS1/E3-V *versus* APP/PS1/E3-6KApoEp	Treatment	0.008	Tukey's HSD method
APP/PS1/E4-V *versus* APP/PS1/E4-6KApoEp	Treatment	0.008	Tukey's HSD method

Table S3 (Supporting information of Fig. 1*D*)

Y-maze test

Alternation

**Two-way ANOVA**	**Factor**	***p* value**
	Genotype	0.002
	Treatment	0.000
	Genotype * Treatment	0.026

**Group comparison**	**Factor**	***p* value**	***post hoc* test**
WT-V *versus* APP/PS1/E2-V	Genotype	0.004	Tukey’s HSD method
WT-V *versus* APP/PS1/E3-V	Genotype	0.003	Tukey’s HSD method
WT-V *versus* APP/PS1/E4-V	Genotype	0.002	Tukey’s HSD method
APP/PS1/E2-V *versus* APP/PS1/E3-V	Genotype	1.000	Tukey’s HSD method
APP/PS1/E2-V *versus* APP/PS1/E4-V	Genotype	1.000	Tukey’s HSD method
WT-6KApoEp *versus* APP/PS1/E2-6KApoEp	Genotype	0.999	Tukey’s HSD method
WT-6KApoEp *versus* APP/PS1/E3-6KApoEp	Genotype	1.000	Tukey’s HSD method
WT-6KApoEp *versus* APP/PS1/E4-6KApoEp	Genotype	0.999	Tukey’s HSD method
APP/PS1/E2-V *versus* APP/PS1/E2-6KApoEp	Treatment	0.025	Tukey’s HSD method
APP/PS1/E3-V *versus* APP/PS1/E3-6KApoEp	Treatment	0.011	Tukey’s HSD method
APP/PS1/E4-V *versus* APP/PS1/E4-6KApoEp	Treatment	0.013	Tukey’s HSD method

Table S4 (Supporting information of Fig. 1, *E* and *F*)

Radial arm water maze test (Errors)

Day 1

Two-way ANOVA

Factor	*p* value
Genotype	0.000
Treatment	0.977
Genotype * Treatment	0.998

Repeated measures ANOVA

Group comparison	Factor	*p* value	*post hoc* test
WT-V *versus* APP/PS1/E2-V	Genotype	0.017	Tukey's HSD method
WT-V *versus* APP/PS1/E3-V	Genotype	0.012	Tukey's HSD method
WT-V *versus* APP/PS1/E4-V	Genotype	0.012	Tukey's HSD method
WT-6KApoEp *versus* APP/PS1/E2-6KApoEp	Genotype	0.012	Tukey's HSD method
WT-6KApoEp *versus* APP/PS1/E3-6KApoEp	Genotype	0.010	Tukey's HSD method
WT-6KApoEp *versus* APP/PS1/E4-6KApoEp	Genotype	0.006	Tukey's HSD method

Day 2

Two-way ANOVA

Factor	*p* value
Genotype	0.000
Treatment	0.000
Genotype * Treatment	0.019

Repeated measures ANOVA

Group comparison	Factor	*p* value	*post hoc* test
WT-V *versus* APP/PS1/E2-V	Genotype	0.000	Tukey's HSD method
WT-V *versus* APP/PS1/E3-V	Genotype	0.000	Tukey's HSD method
WT-V *versus* APP/PS1/E4-V	Genotype	0.000	Tukey's HSD method
WT-6KApoEp *versus* APP/PS1/E2-6KApoEp	Genotype	0.162	Tukey's HSD method
WT-6KApoEp *versus* APP/PS1/E3-6KApoEp	Genotype	0.208	Tukey's HSD method
WT-6KApoEp *versus* APP/PS1/E4-6KApoEp	Genotype	0.110	Tukey's HSD method
WT-V *versus* WT-6KApoEp	Treatment	1.000	Tukey's HSD method
APP/PS1/E2-V *versus* APP/PS1/E2-6KApoEp	Treatment	0.035	Tukey's HSD method
APP/PS1/E3-V *versus* APP/PS1/E3-6KApoEp	Treatment	0.025	Tukey's HSD method
APP/PS1/E4-V *versus* APP/PS1/E4-6KApoEp	Treatment	0.019	Tukey's HSD method

Table S5 (Supporting information of Fig. 1, *G* and *H*)

Radial arm water maze test (Escape latency)

Day 1

Two-way ANOVA

Factor	*p* value
Genotype	0.000
Treatment	0.759
Genotype * Treatment	0.996

Repeated measures ANOVA

Group comparison	Factor	*p* value	*post hoc* test
WT-V *versus* APP/PS1/E2-V	Genotype	0.003	Tukey's HSD method
WT-V *versus* APP/PS1/E3-V	Genotype	0.001	Tukey's HSD method
WT-V *versus* APP/PS1/E4-V	Genotype	0.001	Tukey's HSD method
WT-6KApoEp *versus* APP/PS1/E2-6KApoEp	Genotype	0.023	Tukey's HSD method
WT-6KApoEp *versus* APP/PS1/E3-6KApoEp	Genotype	0.023	Tukey's HSD method
WT-6KApoEp *versus* APP/PS1/E4-6KApoEp	Genotype	0.017	Tukey's HSD method

Day 2

Two-way ANOVA

Factor	*p* value
Genotype	0.000
Treatment	0.000
Genotype * Treatment	0.049

Repeated measures ANOVA

Group comparison	Factor	*p* value	*post hoc* test
WT-V *versus* APP/PS1/E2-V	Genotype	0.000	Tukey's HSD method
WT-V *versus* APP/PS1/E3-V	Genotype	0.000	Tukey's HSD method
WT-V *versus* APP/PS1/E4-V	Genotype	0.000	Tukey's HSD method
WT-6KApoEp *versus* APP/PS1/E2-6KApoEp	Genotype	0.256	Tukey's HSD method
WT-6KApoEp *versus* APP/PS1/E3-6KApoEp	Genotype	0.425	Tukey's HSD method
WT-6KApoEp *versus* APP/PS1/E4-6KApoEp	Genotype	0.126	Tukey's HSD method
WT-V *versus* WT-6KApoEp	Treatment	1.000	Tukey's HSD method
APP/PS1/E2-V *versus* APP/PS1/E2-6KApoEp	Treatment	0.036	Tukey's HSD method
APP/PS1/E3-V *versus* APP/PS1/E3-6KApoEp	Treatment	0.024	Tukey's HSD method
APP/PS1/E4-V *versus* APP/PS1/E4-6KApoEp	Treatment	0.029	Tukey's HSD method

Table S6 (Supporting information of Fig. 3, *A–C*)

Aβ burden

RSC

**Two-way ANOVA**	**Factor**	***p* value**
	Genotype	0.000
	Treatment	0.000
	Genotype * Treatment	0.146

**Group comparison**	**Factor**	***p* value**	***post hoc* test**
APP/PS1/E2-V *versus* APP/PS1/E4-V	Genotype	0.000	Tukey's HSD method
APP/PS1/E3-V *versus* APP/PS1/E4-V	Genotype	0.002	Tukey's HSD method
APP/PS1/E2-6KApoEp *versus* APP/PS1/E4-6KApoEp	Genotype	0.004	Tukey's HSD method
APP/PS1/E3-6KApoEp *versus* APP/PS1/E4-6KApoEp	Genotype	0.112	Tukey's HSD method
APP/PS1/E2-V *versus* APP/PS1/E2-6KApoEp	Treatment	0.000	*t*-test for two samples
APP/PS1/E3-V *versus* APP/PS1/E3-6KApoEp	Treatment	0.000	*t*-test for two samples
APP/PS1/E4-V *versus* APP/PS1/E4-6KApoEp	Treatment	0.000	*t*-test for two samples

H

**Two-way ANOVA**	**Factor**	***p* value**
	Genotype	0.000
	Treatment	0.000
	Genotype * Treatment	0.000

**Group comparison**	**Factor**	***p* value**	***post hoc* test**
APP/PS1/E2-V *versus* APP/PS1/E4-V	Genotype	0.000	Tukey's HSD method
APP/PS1/E3-V *versus* APP/PS1/E4-V	Genotype	0.000	Tukey's HSD method
APP/PS1/E2-6KApoEp *versus* APP/PS1/E4-6KApoEp	Genotype	0.659	Tukey's HSD method
APP/PS1/E3-6KApoEp *versus* APP/PS1/E4-6KApoEp	Genotype	0.004	Tukey's HSD method
APP/PS1/E2-V *versus* APP/PS1/E2-6KApoEp	Treatment	0.000	Tukey's HSD method
APP/PS1/E3-V *versus* APP/PS1/E3-6KApoEp	Treatment	0.000	Tukey's HSD method
APP/PS1/E4-V *versus* APP/PS1/E4-6KApoEp	Treatment	0.000	Tukey's HSD method

EC

**Two-way ANOVA**	**Factor**	***p* value**
	Genotype	0.000
	Treatment	0.000
	Genotype * Treatment	0.000

**Group comparison**	**Factor**	***p* value**	***post hoc* test**
APP/PS1/E2-V *versus* APP/PS1/E4-V	Genotype	0.000	Dunnett's T3 method
APP/PS1/E3-V *versus* APP/PS1/E4-V	Genotype	0.001	Dunnett's T3 method
APP/PS1/E2-6KApoEp *versus* APP/PS1/E4-6KApoEp	Genotype	0.002	Dunnett's T3 method
APP/PS1/E3-6KApoEp *versus* APP/PS1/E4-6KApoEp	Genotype	0.076	Dunnett's T3 method
APP/PS1/E2-V *versus* APP/PS1/E2-6KApoEp	Treatment	0.000	Dunnett's T3 method
APP/PS1/E3-V *versus* APP/PS1/E3-6KApoEp	Treatment	0.000	Dunnett's T3 method
APP/PS1/E4-V *versus* APP/PS1/E4-6KApoEp	Treatment	0.000	Dunnett's T3 method

Table S7 (Supporting information of Fig. 3*D*)

Mean deposit number (< 25 μm)

RSC

**Two-way ANOVA**	**Factor**	***p* value**
	Genotype	0.001
	Treatment	0.000
	Genotype * Treatment	0.211

**Group comparison**	**Factor**	***p* value**	***post hoc* test**
APP/PS1/E2-V *versus* APP/PS1/E4-V	Genotype	0.109	Dunnett's T3 method
APP/PS1/E3-V *versus* APP/PS1/E4-V	Genotype	0.088	Dunnett's T3 method
APP/PS1/E2-6KApoEp *versus* APP/PS1/E4-6KApoEp	Genotype	0.035	Tukey's HSD method
APP/PS1/E3-6KApoEp *versus* APP/PS1/E4-6KApoEp	Genotype	0.298	Tukey's HSD method
APP/PS1/E2-V *versus* APP/PS1/E2-6KApoEp	Treatment	0.000	*t* -test for two samples
APP/PS1/E3-V *versus* APP/PS1/E3-6KApoEp	Treatment	0.000	*t* -test for two samples
APP/PS1/E4-V *versus* APP/PS1/E4-6KApoEp	Treatment	0.004	*t* -test for two samples

H

**Two-way ANOVA**	**Factor**	***p* value**
	Genotype	0.000
	Treatment	0.000
	Genotype * Treatment	0.324

**Group comparison**	**Factor**	***p* value**	***post hoc* test**
APP/PS1/E2-V *versus* APP/PS1/E4-V	Genotype	0.003	Tukey's HSD method
APP/PS1/E3-V *versus* APP/PS1/E4-V	Genotype	0.021	Tukey's HSD method
APP/PS1/E2-6KApoEp *versus* APP/PS1/E4-6KApoEp	Genotype	0.012	Tukey's HSD method
APP/PS1/E3-6KApoEp *versus* APP/PS1/E4-6KApoEp	Genotype	0.046	Tukey's HSD method
APP/PS1/E2-V *versus* APP/PS1/E2-6KApoEp	Treatment	0.010	*t* -test for two samples
APP/PS1/E3-V *versus* APP/PS1/E3-6KApoEp	Treatment	0.001	*t* -test for two samples
APP/PS1/E4-V *versus* APP/PS1/E4-6KApoEp	Treatment	0.000	*t* -test for two samples

EC

**Two-way ANOVA**	**Factor**	***p* value**
	Genotype	0.000
	Treatment	0.000
	Genotype * Treatment	0.171

**Group comparison**	**Factor**	***p* value**	***post hoc* test**
APP/PS1/E2-V *versus* APP/PS1/E4-V	Genotype	0.000	Tukey's HSD method
APP/PS1/E3-V *versus* APP/PS1/E4-V	Genotype	0.001	Tukey's HSD method
APP/PS1/E2-6KApoEp *versus* APP/PS1/E4-6KApoEp	Genotype	0.013	Dunnett's T3 method
APP/PS1/E3-6KApoEp *versus* APP/PS1/E4-6KApoEp	Genotype	0.145	Dunnett's T3 method
APP/PS1/E2-V *versus* APP/PS1/E2-6KApoEp	Treatment	0.002	*t* -test for two samples
APP/PS1/E3-V *versus* APP/PS1/E3-6KApoEp	Treatment	0.000	*t* -test for two samples
APP/PS1/E4-V *versus* APP/PS1/E4-6KApoEp	Treatment	0.002	*t* -test for two samples

Table S8 (Supporting information of Fig. 3*E*)

Mean deposit number (between 25 and 50 μm)

RSC

Two-way ANOVA

Factor	*p* value
Genotype	0.000
Treatment	0.000
Genotype * Treatment	0.037

Group comparison	Factor	*p* value	*post hoc* test
APP/PS1/E2-V *versus* APP/PS1/E4-V	Genotype	0.015	Dunnett's T3 method
APP/PS1/E3-V *versus* APP/PS1/E4-V	Genotype	0.037	Dunnett's T3 method
APP/PS1/E2-6KApoEp *versus* APP/PS1/E4-6KApoEp	Genotype	0.029	Dunnett's T3 method
APP/PS1/E3-6KApoEp *versus* APP/PS1/E4-6KApoEp	Genotype	0.382	Dunnett's T3 method
APP/PS1/E2-V *versus* APP/PS1/E2-6KApoEp	Treatment	0.004	Dunnett's T3 method
APP/PS1/E3-V *versus* APP/PS1/E3-6KApoEp	Treatment	0.001	Dunnett's T3 method
APP/PS1/E4-V *versus* APP/PS1/E4-6KApoEp	Treatment	0.002	Dunnett's T3 method

H

Two-way ANOVA

Factor	*p* value
Genotype	0.000
Treatment	0.000
Genotype * Treatment	0.207

Group comparison	Factor	*p* value	*post hoc* test
APP/PS1/E2-V *versus* APP/PS1/E4-V	Genotype	0.001	Tukey's HSD method
APP/PS1/E3-V *versus* APP/PS1/E4-V	Genotype	0.006	Tukey's HSD method
APP/PS1/E2-6KApoEp *versus* APP/PS1/E4-6KApoEp	Genotype	0.002	Tukey's HSD method
APP/PS1/E3-6KApoEp *versus* APP/PS1/E4-6KApoEp	Genotype	0.018	Tukey's HSD method
APP/PS1/E2-V *versus* APP/PS1/E2-6KApoEp	Treatment	0.006	*t* -test for two samples
APP/PS1/E3-V *versus* APP/PS1/E3-6KApoEp	Treatment	0.000	*t* -test for two samples
APP/PS1/E4-V *versus* APP/PS1/E4-6KApoEp	Treatment	0.000	*t* -test for two samples

EC

Two-way ANOVA

Factor	*p* value
Genotype	0.000
Treatment	0.000
Genotype * Treatment	0.012

Group comparison	Factor	*p* value	*post hoc* test
APP/PS1/E2-V *versus* APP/PS1/E4-V	Genotype	0.000	Tukey's HSD method
APP/PS1/E3-V *versus* APP/PS1/E4-V	Genotype	0.000	Tukey's HSD method
APP/PS1/E2-6KApoEp *versus* APP/PS1/E4-6KApoEp	Genotype	0.000	Tukey's HSD method
APP/PS1/E3-6KApoEp *versus* APP/PS1/E4-6KApoEp	Genotype	0.002	Tukey's HSD method
APP/PS1/E2-V *versus* APP/PS1/E2-6KApoEp	Treatment	0.000	Tukey's HSD method
APP/PS1/E3-V *versus* APP/PS1/E3-6KApoEp	Treatment	0.003	Tukey's HSD method
APP/PS1/E4-V *versus* APP/PS1/E4-6KApoEp	Treatment	0.000	Tukey's HSD method

Table S9 (Supporting information of Fig. 3*F*)

Mean deposit number (> 50 μm)

RSC

Two-way ANOVA

Factor	*p* value
Genotype	0.000
Treatment	0.000
Genotype * Treatment	0.045

Group comparison	Factor	*p* value	*post hoc* test
APP/PS1/E2-V *versus* APP/PS1/E4-V	Genotype	0.000	Tukey's HSD method
APP/PS1/E3-V *versus* APP/PS1/E4-V	Genotype	0.003	Tukey's HSD method
APP/PS1/E2-6KApoEp *versus* APP/PS1/E4-6KApoEp	Genotype	0.223	Tukey's HSD method
APP/PS1/E3-6KApoEp *versus* APP/PS1/E4-6KApoEp	Genotype	0.989	Tukey's HSD method
APP/PS1/E2-V *versus* APP/PS1/E2-6KApoEp	Treatment	0.003	Tukey's HSD method
APP/PS1/E3-V *versus* APP/PS1/E3-6KApoEp	Treatment	0.012	Tukey's HSD method
APP/PS1/E4-V *versus* APP/PS1/E4-6KApoEp	Treatment	0.000	Tukey's HSD method

H

Two-way ANOVA

Factor	*p* value
Genotype	0.000
Treatment	0.000
Genotype * Treatment	0.000

Group comparison	Factor	*p* value	*post hoc* test
APP/PS1/E2-V *versus* APP/PS1/E4-V	Genotype	0.002	Dunnett's T3 method
APP/PS1/E3-V *versus* APP/PS1/E4-V	Genotype	0.002	Dunnett's T3 method
APP/PS1/E2-6KApoEp *versus* APP/PS1/E4-6KApoEp	Genotype	0.102	Dunnett's T3 method
APP/PS1/E3-6KApoEp *versus* APP/PS1/E4-6KApoEp	Genotype	0.003	Dunnett's T3 method
APP/PS1/E2-V *versus* APP/PS1/E2-6KApoEp	Treatment	0.000	Dunnett's T3 method
APP/PS1/E3-V *versus* APP/PS1/E3-6KApoEp	Treatment	0.000	Dunnett's T3 method
APP/PS1/E4-V *versus* APP/PS1/E4-6KApoEp	Treatment	0.000	Dunnett's T3 method

EC

Two-way ANOVA

Factor	*p* value
Genotype	0.000
Treatment	0.000
Genotype * Treatment	0.000

Group comparison	Factor	*p* value	*post hoc* test
APP/PS1/E2-V *versus* APP/PS1/E4-V	Genotype	0.002	Dunnett's T3 method
APP/PS1/E3-V *versus* APP/PS1/E4-V	Genotype	0.006	Dunnett's T3 method
APP/PS1/E2-6KApoEp *versus* APP/PS1/E4-6KApoEp	Genotype	0.130	Dunnett's T3 method
APP/PS1/E3-6KApoEp *versus* APP/PS1/E4-6KApoEp	Genotype	0.262	Dunnett's T3 method
APP/PS1/E2-V *versus* APP/PS1/E2-6KApoEp	Treatment	0.003	Dunnett's T3 method
APP/PS1/E3-V *versus* APP/PS1/E3-6KApoEp	Treatment	0.001	Dunnett's T3 method
APP/PS1/E4-V *versus* APP/PS1/E4-6KApoEp	Treatment	0.001	Dunnett's T3 method

Table S10 (Supporting information of Fig. 4, *A–C*)

Mean CAA number

RSC

Two-way ANOVA	Factor	*p* value
	Genotype	0.000
	Treatment	0.000
	Genotype * Treatment	0.787

Group comparison	Factor	*p* value	*post hoc* test
APP/PS1/E2-V *versus* APP/PS1/E4-V	Genotype	0.010	Tukey's HSD method
APP/PS1/E3-V *versus* APP/PS1/E4-V	Genotype	0.008	Tukey's HSD method
APP/PS1/E2-6KApoEp *versus* APP/PS1/E4-6KApoEp	Genotype	0.068	Tukey's HSD method
APP/PS1/E3-6KApoEp *versus* APP/PS1/E4-6KApoEp	Genotype	0.026	Tukey's HSD method
APP/PS1/E2-V *versus* APP/PS1/E2-6KApoEp	Treatment	0.003	*t* -test for two samples
APP/PS1/E3-V *versus* APP/PS1/E3-6KApoEp	Treatment	0.002	*t* -test for two samples
APP/PS1/E4-V *versus* APP/PS1/E4-6KApoEp	Treatment	0.003	*t* -test for two samples

H

Two-way ANOVA	Factor	*p* value
	Genotype	0.000
	Treatment	0.000
	Genotype * Treatment	0.576

Group comparison	Factor	*p* value	*post hoc* test
APP/PS1/E2-V *versus* APP/PS1/E4-V	Genotype	0.018	Tukey's HSD method
APP/PS1/E3-V *versus* APP/PS1/E4-V	Genotype	0.009	Tukey's HSD method
APP/PS1/E2-6KApoEp *versus* APP/PS1/E4-6KApoEp	Genotype	0.135	Tukey's HSD method
APP/PS1/E3-6KApoEp *versus* APP/PS1/E4-6KApoEp	Genotype	0.057	Tukey's HSD method
APP/PS1/E2-V *versus* APP/PS1/E2-6KApoEp	Treatment	0.002	*t* -test for two samples
APP/PS1/E3-V *versus* APP/PS1/E3-6KApoEp	Treatment	0.003	*t* -test for two samples
APP/PS1/E4-V *versus* APP/PS1/E4-6KApoEp	Treatment	0.001	*t* -test for two samples

EC

Two-way ANOVA	Factor	*p* value
	Genotype	0.000
	Treatment	0.000
	Genotype * Treatment	0.937

Group comparison	Factor	*p* value	*post hoc* test
APP/PS1/E2-V *versus* APP/PS1/E4-V	Genotype	0.021	Tukey's HSD method
APP/PS1/E3-V *versus* APP/PS1/E4-V	Genotype	0.023	Tukey's HSD method
APP/PS1/E2-6KApoEp *versus* APP/PS1/E4-6KApoEp	Genotype	0.010	Tukey's HSD method
APP/PS1/E3-6KApoEp *versus* APP/PS1/E4-6KApoEp	Genotype	0.012	Tukey's HSD method
APP/PS1/E2-V *versus* APP/PS1/E2-6KApoEp	Treatment	0.002	*t* -test for two samples
APP/PS1/E3-V *versus* APP/PS1/E3-6KApoEp	Treatment	0.003	*t* -test for two samples
APP/PS1/E4-V *versus* APP/PS1/E4-6KApoEp	Treatment	0.009	*t* -test for two samples

Table S11 (Supporting information of Fig. 5, *A* and *B*)

TBS-soluble

Aβ1-40 (pM)

Two-way ANOVA

**Factor**	***p* value**
Genotype	0.034
Treatment	0.000
Genotype * Treatment	0.072

**Group comparison**	**Factor**	***p* value**	***post hoc* test**
APP/PS1/E2-V *versus* APP/PS1/E4-V	Genotype	0.071	Tukey's HSD method
APP/PS1/E3-V *versus* APP/PS1/E4-V	Genotype	0.060	Tukey's HSD method
APP/PS1/E2-6KApoEp *versus* APP/PS1/E4-6KApoEp	Genotype	0.893	Tukey's HSD method
APP/PS1/E3-6KApoEp *versus* APP/PS1/E4-6KApoEp	Genotype	0.949	Tukey's HSD method
APP/PS1/E2-V *versus* APP/PS1/E2-6KApoEp	Treatment	0.001	*t* -test for two samples
APP/PS1/E3-V *versus* APP/PS1/E3-6KApoEp	Treatment	0.002	*t* -test for two samples
APP/PS1/E4-V *versus* APP/PS1/E4-6KApoEp	Treatment	0.001	*t* -test for two samples

Aβ1-42 (pM)

Two-way ANOVA

**Factor**	***p* value**
Genotype	0.118
Treatment	0.000
Genotype * Treatment	0.874

**Group comparison**	**Factor**	***p* value**	***post hoc* test**
APP/PS1/E2-V *versus* APP/PS1/E2-6KApoEp	Treatment	0.005	*t* -test for two samples
APP/PS1/E3-V *versus* APP/PS1/E3-6KApoEp	Treatment	0.003	*t* -test for two samples
APP/PS1/E4-V *versus* APP/PS1/E4-6KApoEp	Treatment	0.004	*t* -test for two samples

Table S12 (Supporting information of Fig. 5, *C* and *D*)

Detergent-soluble

Aβ1-40 (nM)

Two-way ANOVA

Factor	*p* value
Genotype	0.642
Treatment	0.000
Genotype * Treatment	0.615

Group comparison	Factor	*p* value	*post hoc* test
APP/PS1/E2-V *versus* APP/PS1/E2-6KApoEp	Treatment	0.004	*t* -test for two samples
APP/PS1/E3-V *versus* APP/PS1/E3-6KApoEp	Treatment	0.004	*t* -test for two samples
APP/PS1/E4-V *versus* APP/PS1/E4-6KApoEp	Treatment	0.003	*t* -test for two samples

Aβ1-42 (nM)

Two-way ANOVA

Factor	*p* value
Genotype	0.567
Treatment	0.000
Genotype * Treatment	0.501

Group comparison	Factor	*p* value	*post hoc* test
APP/PS1/E2-V *versus* APP/PS1/E2-6KApoEp	Treatment	0.001	*t* -test for two samples
APP/PS1/E3-V *versus* APP/PS1/E3-6KApoEp	Treatment	0.000	*t* -test for two samples
APP/PS1/E4-V *versus* APP/PS1/E4-6KApoEp	Treatment	0.003	*t* -test for two samples

Table S13 (Supporting information of Fig. 5, *E* and *F*)

Guanidine-soluble

Aβ1-40 (nM)

Two-way ANOVA	Factor	*p* value
	Genotype	0.001
	Treatment	0.000
	Genotype * Treatment	0.356

Group comparison	Factor	*p* value	*post hoc* test
APP/PS1/E2-V *versus* APP/PS1/E4-V	Genotype	0.032	Tukey's HSD method
APP/PS1/E3-V *versus* APP/PS1/E4-V	Genotype	0.044	Tukey's HSD method
APP/PS1/E2-6KApoEp *versus* APP/PS1/E4-6KApoEp	Genotype	0.248	Dunnett's T3 method
APP/PS1/E3-6KApoEp *versus* APP/PS1/E4-6KApoEp	Genotype	0.041	Dunnett's T3 method
APP/PS1/E2-V *versus* APP/PS1/E2-6KApoEp	Treatment	0.002	*t*-test for two samples
APP/PS1/E3-V *versus* APP/PS1/E3-6KApoEp	Treatment	0.000	*t*-test for two samples
APP/PS1/E4-V *versus* APP/PS1/E4-6KApoEp	Treatment	0.000	*t*-test for two samples

Aβ1-42 (nM)

Two-way ANOVA	Factor	*p* value
	Genotype	0.000
	Treatment	0.000
	Genotype * Treatment	0.632

Group comparison	Factor	*p* value	*post hoc* test
APP/PS1/E2-V *versus* APP/PS1/E4-V	Genotype	0.008	Tukey's HSD method
APP/PS1/E3-V *versus* APP/PS1/E4-V	Genotype	0.016	Tukey's HSD method
APP/PS1/E2-6KApoEp *versus* APP/PS1/E4-6KApoEp	Genotype	0.024	Tukey's HSD method
APP/PS1/E3-6KApoEp *versus* APP/PS1/E4-6KApoEp	Genotype	0.067	Tukey's HSD method
APP/PS1/E2-V *versus* APP/PS1/E2-6KApoEp	Treatment	0.002	*t*-test for two samples
APP/PS1/E3-V *versus* APP/PS1/E3-6KApoEp	Treatment	0.001	*t*-test for two samples
APP/PS1/E4-V *versus* APP/PS1/E4-6KApoEp	Treatment	0.000	*t*-test for two samples

Table S14 (Supporting information of Fig. 6*B*)

APP/actin ratio

**Two-way ANOVA**	**Factor**	***p* value**
	Genotype	0.000
	Treatment	0.000
	Genotype * Treatment	0.673

**Group comparison**	**Factor**	***p* value**	***post hoc* test**
APP/PS1/E2-V *versus* APP/PS1/E4-V	Genotype	0.011	Tukey's HSD method
APP/PS1/E3-V *versus* APP/PS1/E4-V	Genotype	0.011	Tukey's HSD method
APP/PS1/E2-6KApoEp *versus* APP/PS1/E4-6KApoEp	Genotype	0.065	Tukey's HSD method
APP/PS1/E3-6KApoEp *versus* APP/PS1/E4-6KApoEp	Genotype	0.106	Tukey's HSD method
APP/PS1/E2-V *versus* APP/PS1/E2-6KApoEp	Treatment	0.007	*t* -test for two samples
APP/PS1/E3-V *versus* APP/PS1/E3-6KApoEp	Treatment	0.006	*t* -test for two samples
APP/PS1/E4-V *versus* APP/PS1/E4-6KApoEp	Treatment	0.001	*t* -test for two samples

Table S15 (Supporting information of Fig. 6*C*)

App/β-actin mRNA ratio

**Two-way ANOVA**	**Factor**	***p* value**
	Genotype	0.059
	Treatment	0.000
	Genotype * Treatment	0.902

**Group comparison**	**Factor**	***p* value**	***post hoc* test**
APP/PS1/E2-V *versus* APP/PS1/E2-6KApoEp	Treatment	0.036	*t*-test for two samples
APP/PS1/E3-V *versus* APP/PS1/E3-6KApoEp	Treatment	0.031	*t*-test for two samples
APP/PS1/E4-V *versus* APP/PS1/E4-6KApoEp	Treatment	0.014	*t*-test for two samples

Table S16 (Supporting information of Fig. 7*B*)

pC99/actin ratio

Two-way ANOVA	Factor	*p* value
	Genotype	0.000
	Treatment	0.000
	Genotype * Treatment	0.001

Group comparison	Factor	*p* value	*post hoc* test
APP/PS1/E2-V *versus* APP/PS1/E4-V	Genotype	0.000	Tukey's HSD method
APP/PS1/E3-V *versus* APP/PS1/E4-V	Genotype	0.000	Tukey's HSD method
APP/PS1/E2-6KApoEp *versus* APP/PS1/E4-6KApoEp	Genotype	0.815	Tukey's HSD method
APP/PS1/E3-6KApoEp *versus* APP/PS1/E4-6KApoEp	Genotype	0.589	Tukey's HSD method
APP/PS1/E2-V *versus* APP/PS1/E2-6KApoEp	Treatment	0.000	Tukey's HSD method
APP/PS1/E3-V *versus* APP/PS1/E3-6KApoEp	Treatment	0.000	Tukey's HSD method
APP/PS1/E4-V *versus* APP/PS1/E4-6KApoEp	Treatment	0.000	Tukey's HSD method

C99/actin ratio

Two-way ANOVA	Factor	*p* value
	Genotype	0.000
	Treatment	0.000
	Genotype * Treatment	0.046

Group comparison	Factor	*p* value	*post hoc* test
APP/PS1/E2-V *versus* APP/PS1/E4-V	Genotype	0.001	Tukey's HSD method
APP/PS1/E3-V *versus* APP/PS1/E4-V	Genotype	0.000	Tukey's HSD method
APP/PS1/E2-6KApoEp *versus* APP/PS1/E4-6KApoEp	Genotype	0.505	Tukey's HSD method
APP/PS1/E3-6KApoEp *versus* APP/PS1/E4-6KApoEp	Genotype	0.189	Tukey's HSD method
APP/PS1/E2-V *versus* APP/PS1/E2-6KApoEp	Treatment	0.000	Tukey's HSD method
APP/PS1/E3-V *versus* APP/PS1/E3-6KApoEp	Treatment	0.000	Tukey's HSD method
APP/PS1/E4-V *versus* APP/PS1/E4-6KApoEp	Treatment	0.000	Tukey's HSD method

Table S17 (Supporting information of Fig. 7*C*)

Aβ/actin ratio

**Two-way ANOVA**	**Factor**	***p* value**
	Genotype	0.000
	Treatment	0.000
	Genotype * Treatment	0.000

**Group comparison**	**Factor**	***p* value**	***post hoc* test**
APP/PS1/E2-V *versus* APP/PS1/E4-V	Genotype	0.000	Tukey's HSD method
APP/PS1/E3-V *versus* APP/PS1/E4-V	Genotype	0.000	Tukey's HSD method
APP/PS1/E2-6KApoEp *versus* APP/PS1/E4-6KApoEp	Genotype	0.340	Tukey's HSD method
APP/PS1/E3-6KApoEp *versus* APP/PS1/E4-6KApoEp	Genotype	0.086	Tukey's HSD method
APP/PS1/E2-V *versus* APP/PS1/E2-6KApoEp	Treatment	0.000	Tukey's HSD method
APP/PS1/E3-V *versus* APP/PS1/E3-6KApoEp	Treatment	0.000	Tukey's HSD method
APP/PS1/E4-V *versus* APP/PS1/E4-6KApoEp	Treatment	0.000	Tukey's HSD method

Table S18 (Supporting information of Fig. 7*D*)

Aβ oligomer (pM)

Two-way ANOVA

Factor	*p* value
Genotype	0.065
Treatment	0.000
Genotype * Treatment	0.727

**Group comparison**	**Factor**	***p* value**	***post hoc* test**
APP/PS1/E2-V *versus* APP/PS1/E2-6KApoEp	Treatment	0.004	*t* -test for two samples
APP/PS1/E3-V *versus* APP/PS1/E3-6KApoEp	Treatment	0.000	*t* -test for two samples
APP/PS1/E4-V *versus* APP/PS1/E4-6KApoEp	Treatment	0.004	*t* -test for two samples

Table S19 (Supporting information of Fig. 7*E*)

BACE1/actin ratio

Two-way ANOVA

**Factor**	***p* value**
Genotype	0.532
Treatment	0.822
Genotype * Treatment	0.626

Table S20 (Supporting information of Fig. 8*B*)

pp44/42/tp44/42 ratio (Upper)

Two-way ANOVA

Factor	*p* value
Genotype	0.067
Treatment	0.000
Genotype * Treatment	0.001

Group comparison	Factor	*p* value	*post hoc* test
APP/PS1/E2-V *versus* APP/PS1/E4-V	Genotype	0.778	Tukey's HSD
APP/PS1/E3-V *versus* APP/PS1/E4-V	Genotype	0.601	Tukey's HSD
APP/PS1/E2-6KApoEp *versus* APP/PS1/E4-6KApoEp	Genotype	0.001	Tukey's HSD
APP/PS1/E3-6KApoEp *versus* APP/PS1/E4-6KApoEp	Genotype	0.037	Tukey's HSD
APP/PS1/E2-V *versus* APP/PS1/E2-6KApoEp	Treatment	0.000	Tukey's HSD
APP/PS1/E3-V *versus* APP/PS1/E3-6KApoEp	Treatment	0.000	Tukey's HSD
APP/PS1/E4-V *versus* APP/PS1/E4-6KApoEp	Treatment	0.000	Tukey's HSD

pp44/42/tp44/42 ratio (Lower)

Two-way ANOVA

Factor	*p* value
Genotype	0.000
Treatment	0.000
Genotype * Treatment	0.000

Group comparison	Factor	*p* value	*post hoc* test
APP/PS1/E2-V *versus* APP/PS1/E4-V	Genotype	1.000	Dunnett's T3
APP/PS1/E3-V *versus* APP/PS1/E4-V	Genotype	1.000	Dunnett's T3
APP/PS1/E2-6KApoEp *versus* APP/PS1/E4-6KApoEp	Genotype	0.006	Dunnett's T3
APP/PS1/E3-6KApoEp *versus* APP/PS1/E4-6KApoEp	Genotype	0.077	Dunnett's T3
APP/PS1/E2-V *versus* APP/PS1/E2-6KApoEp	Treatment	0.000	Dunnett's T3
APP/PS1/E3-V *versus* APP/PS1/E3-6KApoEp	Treatment	0.000	Dunnett's T3
APP/PS1/E4-V *versus* APP/PS1/E4-6KApoEp	Treatment	0.000	Dunnett's T3

Table S21 (Supporting information of Fig. 8*C*)

pp38/tp38 ratio

**Two-way ANOVA**	**Factor**	***p* value**
	Genotype	0.873
	Treatment	0.000
	Genotype * Treatment	0.864

**Group comparison**	**Factor**	***p* value**	***post hoc* test**
APP/PS1/E2-V *versus* APP/PS1/E2-6KApoEp	Treatment	0.000	*t* -test for two samples
APP/PS1/E3-V *versus* APP/PS1/E3-6KApoEp	Treatment	0.001	*t* -test for two samples
APP/PS1/E4-V *versus* APP/PS1/E4-6KApoEp	Treatment	0.001	*t* -test for two samples

Table S22 (Supporting information of Fig. 8*D*)

DLK/actin ratio

Two-way ANOVA

Factor	*p* value
Genotype	0.248
Treatment	0.000
Genotype * Treatment	0.604

**Group comparison**	**Factor**	***p* value**	***post hoc* test**
APP/PS1/E2-V *versus* APP/PS1/E2-6KApoEp	Treatment	0.000	*t* -test for two samples
APP/PS1/E3-V *versus* APP/PS1/E3-6KApoEp	Treatment	0.000	*t* -test for two samples
APP/PS1/E4-V *versus* APP/PS1/E4-6KApoEp	Treatment	0.000	*t* -test for two samples

Table S23 (Supporting information of Fig. 10*D*)

6KApoEp with mouse plasma (h)

	p value
**One-way ANOVA**	0.158

Table S24 (Supporting information of Fig. 1, *A* and *B*)

Object recognition test

WT-V

	Training	Retention test
1	60.00	55.56
2	36.36	72.73
3	45.45	70.00
4	60.00	72.73
5	56.25	50.00
6	41.67	62.50
7	45.45	70.00
8	50.00	62.50
Mean	49.40	64.50
S.D.	8.73	8.40

WT-6KApoEp

	Training	Retention test
1	37.50	55.56
2	53.85	72.73
3	53.85	64.29
4	53.85	58.82
5	53.85	75.00
6	57.14	75.00
7	60.00	75.00
8	37.50	60.00
Mean	50.94	67.05
S.D.	8.58	8.27

APP/PS1/E2-V

	Training	Retention test
1	55.56	44.44
2	53.85	60.00
3	50.00	57.14
4	56.25	61.54
5	58.33	45.45
6	36.36	40.00
7	40.00	50.00
8	53.85	42.86
Mean	50.52	50.18
S.D.	8.04	8.34

APP/PS1/E2-6KApoEp

	Training	Retention test
1	42.86	62.50
2	57.14	75.00
3	50.00	69.23
4	57.14	66.67
5	33.33	57.14
6	53.85	62.50
7	62.50	71.43
8	50.00	50.00
Mean	50.85	64.31
S.D.	9.23	8.08

APP/PS1/E3-V

	Training	Retention test
1	54.55	40.00
2	42.86	53.33
3	57.14	40.00
4	36.36	54.55
5	50.00	50.00
6	40.00	60.00
7	54.55	56.25
8	62.50	40.00
Mean	49.74	49.27
S.D.	9.14	8.16

APP/PS1/E3-6KApoEp

	Training	Retention test
1	50.00	71.43
2	40.00	57.14
3	46.15	73.33
4	58.33	68.75
5	58.33	58.82
6	57.14	73.33
7	40.00	50.00
8	42.86	64.71
Mean	49.10	64.69
S.D.	8.01	8.61

APP/PS1/E4-V

	Training	Retention test
1	50.00	46.15
2	61.54	53.85
3	58.33	46.15
4	41.67	57.14
5	40.00	66.67
6	50.00	41.67
7	50.00	42.86
8	60.00	50.00
Mean	51.44	50.56
S.D.	8.07	8.38

APP/PS1/E4-6KApoEp

	Training	Retention test
1	57.14	69.23
2	43.75	60.00
3	58.33	62.50
4	52.94	69.23
5	43.75	50.00
6	46.15	66.67
7	35.71	78.57
8	57.14	71.43
Mean	49.37	65.95
S.D.	8.23	8.56

Table S25 (Supporting information of Fig. 1*C*)

Y-maze test

WT-V

	Number of arms entered
1	27
2	25
3	20
4	29
5	27
6	40
7	30
8	29
Mean	28.38
S.D.	5.66

WT-6KApoEp

	Number of arms entered
1	27
2	35
3	27
4	29
5	23
6	28
7	21
8	34
Mean	28.00
S.D.	4.81

APP/PS1/E2-V

	Number of arms entered
1	43
2	36
3	39
4	42
5	29
6	34
7	44
8	36
Mean	37.88
S.D.	5.11

APP/PS1/E2-6KApoEp

	Number of arms entered
1	31
2	21
3	32
4	32
5	30
6	26
7	32
8	25
Mean	28.63
S.D.	4.14

APP/PS1/E3-V

	Number of arms entered
1	38
2	28
3	40
4	42
5	42
6	30
7	40
8	36
Mean	37.00
S.D.	5.35

APP/PS1/E3-6KApoEp

	Number of arms entered
1	22
2	26
3	27
4	28
5	35
6	22
7	32
8	28
Mean	27.50
S.D.	4.47

APP/PS1/E4-V

	Number of arms entered
1	36
2	31
3	50
4	36
5	34
6	40
7	32
8	36
Mean	36.88
S.D.	5.99

APP/PS1/E4-6KApoEp

	Number of arms entered
1	25
2	22
3	26
4	29
5	25
6	35
7	25
8	32
Mean	27.38
S.D.	4.31

Table S26 (Supporting information of Fig. 1*D*)

Y-maze test

WT-V

	Alternation
1	60.00
2	69.57
3	66.67
4	62.96
5	48.00
6	57.89
7	60.71
8	59.26
Mean	60.63
S.D.	6.45

WT-6KApoEp

	Alternation
1	64.00
2	57.58
3	56.00
4	66.67
5	61.90
6	50.00
7	68.42
8	59.38
Mean	60.49
S.D.	6.04

APP/PS1/E2-V

	Alternation
1	43.90
2	52.94
3	48.65
4	55.00
5	37.04
6	50.00
7	45.24
8	52.94
Mean	48.21
S.D.	5.93

APP/PS1/E2-6KApoEp

	Alternation
1	62.07
2	52.63
3	56.67
4	60.00
5	67.86
6	50.00
7	60.00
8	60.87
Mean	58.76
S.D.	5.60

APP/PS1/E3-V

	Alternation
1	36.11
2	53.85
3	50.00
4	45.00
5	47.50
6	57.14
7	50.00
8	44.12
Mean	47.96
S.D.	6.45

APP/PS1/E3-6KApoEp

	Alternation
1	60.00
2	54.17
3	56.00
4	61.54
5	51.52
6	70.00
7	56.67
8	65.38
Mean	59.41
S.D.	6.11

APP/PS1/E4-V

	Alternation
1	47.06
2	51.72
3	35.42
4	41.18
5	53.13
6	47.37
7	50.00
8	52.94
Mean	47.35
S.D.	6.23

APP/PS1/E4-6KApoEp

	Alternation
1	56.52
2	50.00
3	70.83
4	62.96
5	60.87
6	51.52
7	56.52
8	60.00
Mean	58.65
S.D.	6.64

Table S27 (Supporting information of Fig. 1*E*)

Radial arm water maze test (Errors)

WT-V

	Day 1					Day 2				
	**1**	**2**	**3**	**4**	**5**	**6**	**7**	**8**	**9**	**10**
1	9.00	3.67	6.00	6.67	2.67	6.67	5.67	4.00	6.00	5.00
2	6.67	5.67	7.33	4.00	4.33	4.33	6.00	4.33	2.67	4.33
3	9.67	9.00	4.00	6.00	2.67	6.33	5.33	2.67	3.00	2.33
4	4.33	5.33	2.67	4.00	3.33	9.33	6.00	2.67	2.67	4.33
5	6.33	4.33	4.33	2.67	4.67	8.00	3.33	4.33	2.67	2.67
6	6.33	5.67	4.67	7.00	7.00	5.67	8.00	4.33	5.33	1.67
7	5.00	5.33	4.67	4.67	7.00	5.33	7.33	5.67	2.33	2.67
8	7.00	7.67	8.00	3.67	3.00	5.67	5.33	7.00	3.67	2.67
Mean	6.79	5.83	5.21	4.83	4.33	6.42	5.88	4.38	3.54	3.21
S.D.	1.81	1.73	1.78	1.55	1.80	1.59	1.40	1.44	1.38	1.18

APP/PS1/E2-V

	Day 1					Day 2				
	**1**	**2**	**3**	**4**	**5**	**6**	**7**	**8**	**9**	**10**
1	5.67	4.33	7.00	6.00	4.67	4.33	5.00	5.33	5.67	8.00
2	6.67	8.33	5.67	5.33	6.67	8.00	8.00	10.33	6.33	8.67
3	7.00	7.67	9.33	7.00	4.33	9.33	8.00	8.33	10.33	9.33
4	7.33	9.33	3.67	4.00	4.33	9.00	7.00	5.67	9.00	5.33
5	8.33	6.00	4.33	10.00	8.00	7.67	8.67	7.33	4.33	7.00
6	9.33	6.67	5.67	8.33	9.33	10.67	7.67	9.67	8.33	7.00
7	11.00	10.00	10.33	10.00	7.67	9.00	10.33	6.67	7.33	4.67
8	10.67	8.33	6.00	6.00	8.00	6.00	5.00	7.67	6.00	4.67
Mean	8.25	7.58	6.50	7.08	6.63	8.00	7.46	7.63	7.17	6.83
S.D.	1.93	1.85	2.31	2.19	1.95	2.02	1.80	1.78	1.97	1.80

APP/PS1/E3-V

	Day 1					Day 2				
	**1**	**2**	**3**	**4**	**5**	**6**	**7**	**8**	**9**	**10**
1	10.33	8.00	5.67	5.67	7.00	7.33	7.67	4.00	6.67	5.00
2	8.67	7.00	4.67	7.00	4.33	5.33	10.33	9.67	6.00	8.33
3	6.33	5.33	4.33	4.00	9.00	7.00	6.67	7.33	5.33	9.33
4	8.33	7.67	9.00	8.00	7.00	7.33	7.67	8.33	3.67	9.33
5	5.67	8.33	10.00	8.33	8.67	8.33	11.67	7.33	7.33	7.00
6	9.00	6.33	6.00	6.33	7.00	8.33	7.67	6.00	8.33	5.67
7	8.67	7.00	7.67	8.67	6.33	10.33	6.67	7.33	6.67	8.67
8	9.33	6.67	7.33	7.33	9.00	11.33	6.00	7.00	9.33	3.33
Mean	8.29	7.04	6.83	6.92	7.29	8.17	8.04	7.13	6.67	7.08
S.D.	1.55	0.97	2.02	1.55	1.59	1.91	1.96	1.65	1.75	2.22

APP/PS1/E4-V

	Day 1					Day 2				
	**1**	**2**	**3**	**4**	**5**	**6**	**7**	**8**	**9**	**10**
1	6.00	4.00	4.67	4.00	6.00	7.67	6.67	5.00	5.33	4.33
2	6.67	6.00	6.00	6.67	5.00	9.67	7.00	8.00	9.67	8.67
3	6.00	5.67	5.33	6.00	11.67	9.33	6.67	7.00	11.00	7.67
4	9.67	6.33	7.67	5.00	6.67	4.33	5.67	8.33	3.33	4.33
5	8.00	9.33	5.67	6.00	7.33	9.33	7.33	9.00	6.67	9.67
6	8.33	8.33	10.00	7.67	7.33	8.00	9.33	7.67	4.33	6.67
7	10.00	8.00	9.00	9.33	7.67	11.33	9.67	6.33	9.67	7.33
8	11.00	10.67	7.00	8.33	7.00	7.00	9.33	9.67	6.67	9.67
Mean	8.21	7.29	6.92	6.63	7.33	8.33	7.71	7.63	7.08	7.29
S.D.	1.90	2.18	1.87	1.76	1.95	2.11	1.52	1.50	2.77	2.11

Table S28 (Supporting information of Fig. 1*F*)

Radial arm water maze test (Errors)

WT-6KApoEp

	Day 1					Day 2				
	**1**	**2**	**3**	**4**	**5**	**6**	**7**	**8**	**9**	**10**
1	5.67	5.00	4.67	5.33	5.33	9.33	4.67	3.67	4.00	2.33
2	4.00	7.33	5.33	7.00	7.00	6.67	4.67	3.67	2.00	5.67
3	6.67	5.67	3.67	2.33	3.00	5.33	4.00	3.67	2.33	2.67
4	9.33	7.00	7.67	6.67	6.00	5.67	3.67	7.33	6.67	2.67
5	8.33	3.67	3.67	2.67	4.33	5.33	5.67	3.00	2.67	2.67
6	3.67	3.33	3.00	4.33	1.67	5.67	5.67	5.67	3.67	2.67
7	8.67	8.33	8.00	5.00	2.33	6.00	7.00	2.00	5.00	4.67
8	8.67	8.00	5.67	3.33	5.00	7.67	5.67	4.67	4.33	5.00
Mean	6.88	6.04	5.21	4.58	4.33	6.46	5.13	4.21	3.83	3.54
S.D.	2.22	1.92	1.85	1.74	1.86	1.40	1.08	1.66	1.54	1.33

APP/PS1/E2-6KApoEp

	Day 1					Day 2				
	**1**	**2**	**3**	**4**	**5**	**6**	**7**	**8**	**9**	**10**
1	4.67	4.33	4.33	3.67	9.33	10.00	7.00	7.00	5.00	7.00
2	9.00	7.33	7.67	10.67	5.33	9.00	9.00	5.33	6.67	4.00
3	9.67	7.00	4.33	5.33	4.33	6.33	5.00	7.67	6.67	2.67
4	8.33	8.33	7.33	8.67	7.33	3.00	6.00	5.00	3.00	4.33
5	7.33	9.33	7.67	5.00	3.33	5.67	5.67	4.33	5.67	5.67
6	9.33	10.67	6.33	6.00	6.00	11.33	9.00	9.00	6.33	3.00
7	9.00	5.67	7.33	8.33	7.33	9.67	5.00	4.00	2.67	5.33
8	10.00	9.67	10.00	5.00	6.67	7.00	5.00	3.67	2.33	5.00
Mean	8.42	7.79	6.88	6.58	6.21	7.75	6.46	5.75	4.79	4.63
S.D.	1.73	2.13	1.88	2.38	1.89	2.74	1.71	1.93	1.85	1.43

APP/PS1/E3-6KApoEp

	Day 1					Day 2				
	**1**	**2**	**3**	**4**	**5**	**6**	**7**	**8**	**9**	**10**
1	8.67	8.00	8.00	7.33	3.67	7.33	8.00	6.33	6.00	4.00
2	6.33	8.33	5.33	6.67	6.67	5.00	5.33	3.33	4.33	6.00
3	6.00	4.00	8.33	5.67	5.33	5.67	5.33	7.00	6.67	5.67
4	9.00	8.67	5.67	4.33	8.00	9.00	5.67	5.00	3.00	5.33
5	10.67	10.67	7.67	8.33	8.33	9.33	8.33	6.00	5.33	2.67
6	10.33	7.33	5.67	6.00	5.33	9.00	4.00	2.67	4.00	4.33
7	10.00	8.67	6.33	6.67	7.67	9.33	4.00	6.33	5.33	6.67
8	10.33	5.67	5.00	8.00	6.33	9.33	6.00	6.00	5.33	4.67
Mean	8.92	7.67	6.50	6.63	6.42	8.00	5.83	5.33	5.00	4.92
S.D.	1.83	2.04	1.31	1.30	1.59	1.78	1.61	1.55	1.17	1.27

APP/PS1/E4-6KApoEp

	Day 1					Day 2				
	**1**	**2**	**3**	**4**	**5**	**6**	**7**	**8**	**9**	**10**
1	9.67	4.67	2.33	6.67	5.67	7.67	5.33	6.33	6.33	5.33
2	10.33	7.33	8.33	6.67	9.67	4.33	8.00	3.00	4.00	4.33
3	6.67	10.33	7.67	5.00	4.33	7.33	4.33	7.00	4.00	6.00
4	7.33	8.67	9.00	6.00	6.67	8.00	6.67	6.33	7.33	5.00
5	10.33	9.33	7.67	7.67	4.33	7.67	8.33	6.00	6.00	6.67
6	8.00	8.33	7.33	8.00	6.00	8.67	4.33	6.33	6.67	3.00
7	10.00	6.67	8.00	4.00	8.67	7.67	8.33	3.33	3.67	5.33
8	10.00	9.00	4.00	7.00	5.67	9.33	8.33	4.33	4.33	3.33
Mean	9.04	8.04	6.79	6.38	6.38	7.58	6.71	5.33	5.29	4.88
S.D.	1.47	1.78	2.34	1.34	1.91	1.47	1.80	1.54	1.44	1.26

Table S29 (Supporting information of Fig. 1*G*)

Radial arm water maze test (Escape latency)

WT-V

	Day 1					Day 2				
	**1**	**2**	**3**	**4**	**5**	**6**	**7**	**8**	**9**	**10**
1	49.67	21.67	43.33	52.00	40.00	43.67	51.67	26.33	32.33	35.67
2	34.00	31.33	41.67	27.67	39.67	30.67	40.33	30.00	24.67	35.67
3	49.00	49.00	24.00	48.67	20.67	47.00	34.67	20.00	18.67	25.00
4	39.33	42.00	20.00	26.33	38.00	50.00	40.67	20.67	26.00	19.33
5	36.00	48.33	37.33	22.33	37.00	44.00	23.67	30.00	24.00	16.67
6	38.00	46.67	33.33	41.33	47.00	40.33	36.67	42.33	39.67	34.67
7	57.00	36.33	39.33	24.67	29.67	36.00	34.67	43.00	20.00	13.67
8	44.33	38.67	41.67	33.00	21.67	32.00	30.67	37.00	30.00	24.67
Mean	43.42	39.25	35.08	34.50	34.21	40.46	36.63	31.17	26.92	25.67
S.D.	7.98	9.42	8.71	11.43	9.34	7.01	8.17	8.94	6.88	8.84

APP/PS1/E2-V

	Day 1					Day 2				
	**1**	**2**	**3**	**4**	**5**	**6**	**7**	**8**	**9**	**10**
1	55.00	53.67	54.00	46.67	23.33	39.00	36.67	34.33	42.67	53.33
2	40.33	46.33	40.67	26.00	57.67	49.67	58.00	57.67	35.33	56.33
3	51.67	44.67	47.33	40.00	27.00	53.00	52.33	44.00	52.33	49.33
4	48.00	46.33	33.67	36.00	45.33	57.33	32.33	29.00	50.67	36.33
5	55.00	37.33	23.33	56.67	42.33	43.33	51.00	43.67	32.33	32.00
6	55.00	46.33	46.67	47.33	49.33	54.67	41.33	49.67	57.00	37.33
7	55.00	52.67	52.00	45.67	41.00	57.33	57.33	36.67	32.67	36.00
8	55.00	46.67	29.33	32.67	39.67	36.33	32.67	39.33	41.67	33.67
Mean	51.88	46.75	40.88	41.38	40.71	48.83	45.21	41.79	43.08	41.79
S.D.	5.31	5.02	11.11	9.70	11.19	8.28	10.73	9.06	9.43	9.61

APP/PS1/E3-V

	Day 1					Day 2				
	**1**	**2**	**3**	**4**	**5**	**6**	**7**	**8**	**9**	**10**
1	51.67	46.33	39.00	33.33	33.00	42.67	43.67	25.67	43.33	30.67
2	48.33	40.00	40.67	38.67	29.00	34.67	59.33	52.33	37.33	41.67
3	35.67	30.33	39.33	32.00	53.67	43.33	46.67	45.67	27.67	46.67
4	55.00	46.00	54.00	40.00	40.67	60.00	36.00	49.33	45.67	50.33
5	48.00	53.67	54.33	54.00	47.33	48.67	59.00	42.00	35.67	35.67
6	55.00	55.00	40.33	47.33	43.67	58.33	48.33	44.33	48.67	34.67
7	55.00	51.00	45.67	49.33	42.67	50.67	45.67	43.33	34.67	39.67
8	51.67	49.33	38.00	46.67	45.00	50.67	39.00	45.33	54.67	30.67
Mean	50.04	46.46	43.92	42.67	41.88	48.63	47.21	43.50	40.96	38.75
S.D.	6.47	8.07	6.73	7.88	7.82	8.37	8.41	7.94	8.72	7.20

APP/PS1/E4-V

	Day 1					Day 2				
	**1**	**2**	**3**	**4**	**5**	**6**	**7**	**8**	**9**	**10**
1	56.67	46.00	40.33	35.00	44.67	55.00	55.67	49.00	48.00	31.67
2	40.00	40.67	39.33	45.33	35.67	55.00	42.00	38.33	48.67	47.33
3	43.67	51.67	29.33	38.33	54.00	51.67	41.67	40.33	50.00	32.67
4	56.67	38.00	49.67	45.33	40.00	54.33	36.33	56.67	30.67	36.33
5	52.67	53.33	36.67	49.33	44.33	51.67	44.33	49.33	40.00	53.67
6	54.00	45.00	54.00	33.00	33.33	45.33	54.67	40.67	32.00	38.33
7	49.33	34.67	50.33	48.33	53.33	52.33	52.00	41.33	52.67	38.67
8	56.67	55.67	40.33	48.33	40.67	31.00	53.33	48.67	33.67	47.00
Mean	51.21	45.63	42.50	42.88	43.25	49.54	47.50	45.54	41.96	40.71
S.D.	6.38	7.56	8.21	6.48	7.50	8.11	7.28	6.33	8.95	7.81

Table S30 (Supporting information of Fig. 1*H*)

Radial arm water maze test (Escape latency)

WT-6KApoEp

	Day 1					Day 2				
	**1**	**2**	**3**	**4**	**5**	**6**	**7**	**8**	**9**	**10**
1	35.67	35.00	31.00	40.67	41.67	50.67	33.67	39.00	22.67	26.00
2	36.00	42.67	36.33	48.00	46.00	50.67	37.33	38.67	22.67	26.00
3	32.67	42.67	24.67	26.67	21.00	33.00	28.67	20.67	16.33	25.67
4	43.67	49.33	49.00	42.00	39.33	49.67	23.00	45.00	41.33	29.67
5	59.00	27.00	34.00	21.00	25.67	35.33	33.67	23.67	15.00	18.67
6	45.00	34.00	16.00	34.00	33.00	30.33	37.33	33.67	21.00	11.67
7	49.67	49.67	50.33	38.00	22.67	37.33	57.67	16.33	39.00	38.67
8	42.00	33.33	38.33	25.00	40.00	39.00	33.33	30.33	41.00	25.67
Mean	42.96	39.21	34.96	34.42	33.67	40.75	35.58	30.92	27.38	25.25
S.D.	8.58	8.14	11.52	9.43	9.52	8.36	10.09	10.01	11.18	7.83

APP/PS1/E2-6KApoEp

	Day 1					Day 2				
	**1**	**2**	**3**	**4**	**5**	**6**	**7**	**8**	**9**	**10**
1	42.33	36.33	25.67	22.67	53.33	56.00	45.00	40.67	32.33	34.33
2	54.00	43.00	51.33	50.00	30.00	49.67	49.00	27.33	46.00	29.67
3	57.67	52.00	34.33	42.33	28.67	40.33	30.67	41.33	38.67	17.00
4	54.00	53.33	52.67	52.00	40.67	28.33	35.00	33.33	20.00	29.00
5	36.33	50.67	43.67	41.00	33.67	30.33	44.33	51.00	37.33	44.00
6	51.00	57.67	48.67	40.00	48.00	53.00	41.67	36.00	49.33	41.00
7	48.33	52.33	44.00	40.33	43.67	53.00	28.00	28.67	22.00	29.67
8	57.00	43.00	60.00	23.00	36.33	45.00	40.00	27.00	26.67	33.67
Mean	50.08	48.54	45.04	38.92	39.29	44.46	39.21	35.67	34.04	32.29
S.D.	7.46	7.05	10.87	10.89	8.73	10.58	7.35	8.38	10.75	8.26

APP/PS1/E3-6KApoEp

	Day 1					Day 2				
	**1**	**2**	**3**	**4**	**5**	**6**	**7**	**8**	**9**	**10**
1	54.00	50.33	54.33	42.33	23.33	44.00	53.33	32.00	30.00	26.67
2	42.67	40.67	31.00	32.67	32.67	34.00	39.67	27.67	21.33	29.67
3	38.00	43.33	56.67	33.67	25.67	35.00	35.00	46.33	38.67	28.67
4	54.67	46.00	42.33	35.67	54.00	50.67	40.00	35.00	43.33	35.67
5	53.00	58.33	47.00	41.67	33.33	52.00	59.00	31.33	29.67	16.00
6	56.67	50.00	44.33	50.67	42.67	50.67	35.00	21.67	27.33	41.00
7	57.67	54.00	43.67	33.33	48.33	56.00	34.67	41.67	22.67	33.67
8	57.67	33.00	38.00	54.67	43.00	46.67	37.67	36.00	34.67	25.00
Mean	51.79	46.96	44.67	40.58	37.88	46.13	41.79	33.96	30.96	29.54
S.D.	7.38	8.00	8.29	8.38	10.87	8.01	9.24	7.74	7.59	7.55

APP/PS1/E4-6KApoEp

	Day 1					Day 2				
	**1**	**2**	**3**	**4**	**5**	**6**	**7**	**8**	**9**	**10**
1	56.00	26.67	23.67	43.33	33.33	52.33	39.67	48.00	37.33	35.33
2	56.00	50.67	52.67	54.33	48.67	35.00	50.67	25.00	29.67	40.33
3	48.67	57.00	44.67	33.33	46.67	58.33	22.67	41.33	25.33	35.33
4	44.00	47.67	56.00	41.00	40.00	50.00	42.00	51.67	37.67	26.67
5	54.67	48.33	46.00	47.00	44.67	49.33	53.00	34.67	45.00	34.67
6	38.00	48.33	43.33	46.67	36.67	44.00	30.33	47.33	41.33	15.00
7	58.00	40.33	43.33	32.67	49.00	40.00	43.00	27.00	16.67	31.67
8	54.67	44.33	36.33	39.00	30.67	44.00	47.67	26.67	27.33	32.33
Mean	51.25	45.42	43.25	42.17	41.21	46.63	41.13	37.71	32.54	31.42
S.D.	7.06	8.96	9.94	7.30	7.10	7.38	10.27	10.78	9.42	7.69

Table S31 (Supporting information of Fig. 3*D*)

Mean deposit number (< 25 µm)

APP/PS1/E2-V

	RSC	H	EC
1	227	146	149
2	209	186	142
3	164	198	144
4	188	143	177
5	203	210	206
6	190	252	205
7	180	251	171
8	215	235	241
Mean	197.00	202.63	179.38
S.D.	20.40	42.99	35.48

APP/PS1/E2-6KApoEp

	RSC	H	EC
1	123	158	121
2	141	131	129
3	109	136	120
4	109	156	94
5	179	175	140
6	165	171	140
7	121	173	131
8	149	124	106
Mean	137.00	153.00	122.63
S.D.	25.99	20.20	16.12

APP/PS1/E3-V

	RSC	H	EC
1	191	180	193
2	186	200	209
3	183	192	209
4	174	218	211
5	218	227	176
6	194	237	174
7	191	266	186
8	219	225	236
Mean	194.50	218.13	199.25
S.D.	16.04	27.36	20.95

APP/PS1/E3-6KApoEp

	RSC	H	EC
1	167	169	168
2	130	148	147
3	134	138	126
4	149	196	159
5	150	190	132
6	128	125	134
7	192	157	175
8	147	159	164
Mean	149.63	160.25	150.63
S.D.	21.39	24.32	18.47

APP/PS1/E4-V

	RSC	H	EC
1	175	222	271
2	255	283	323
3	176	236	303
4	201	241	176
5	312	283	262
6	315	337	296
7	287	269	287
8	272	307	347
Mean	249.13	272.25	283.13
S.D.	57.87	38.65	51.15

APP/PS1/E4-6KApoEp

	RSC	H	EC
1	162	170	131
2	144	175	154
3	150	176	175
4	182	156	144
5	187	204	194
6	157	208	262
7	187	237	227
8	161	198	236
Mean	166.25	190.50	190.38
S.D.	16.90	26.10	47.51

Table S32 (Supporting information of Fig. 3*E*)

Mean deposit number (between 25 and 50 µm)

APP/PS1/E2-V

	RSC	H	EC
1	100	52	82
2	90	67	61
3	102	70	59
4	79	45	69
5	62	67	79
6	105	69	94
7	64	108	78
8	69	106	78
Mean	83.88	73.00	75.00
S.D.	17.69	22.79	11.54

APP/PS1/E2-6KApoEp

	RSC	H	EC
1	61	41	41
2	59	45	33
3	40	48	33
4	39	65	27
5	44	30	36
6	43	50	49
7	38	42	54
8	52	27	36
Mean	47.00	43.50	38.63
S.D.	9.13	11.89	8.96

APP/PS1/E3-V

	RSC	H	EC
1	81	66	79
2	77	63	80
3	85	76	83
4	96	94	60
5	108	97	60
6	114	98	72
7	75	95	79
8	90	65	76
Mean	90.75	81.75	73.63
S.D.	14.30	15.75	8.99

APP/PS1/E3-6KApoEp

	RSC	H	EC
1	61	53	46
2	53	32	43
3	66	63	44
4	61	53	60
5	63	65	39
6	37	40	43
7	54	39	48
8	54	48	55
Mean	56.13	49.13	47.25
S.D.	9.08	11.68	6.96

APP/PS1/E4-V

	RSC	H	EC
1	101	106	143
2	160	124	133
3	95	90	156
4	107	108	92
5	143	100	123
6	138	142	125
7	148	113	135
8	161	137	138
Mean	131.63	115.00	130.63
S.D.	26.70	18.04	18.74

APP/PS1/E4-6KApoEp

	RSC	H	EC
1	83	68	55
2	53	53	73
3	56	69	79
4	72	50	44
5	82	84	88
6	58	61	70
7	63	76	98
8	88	72	95
Mean	69.38	66.63	75.25
S.D.	13.71	11.46	18.91

Table S33 (Supporting information of Fig. 3*F*)

Mean deposit number (> 50 µm)

APP/PS1/E2-V

	RSC	H	EC
1	26	33	21
2	36	39	24
3	21	40	29
4	18	41	24
5	29	43	34
6	24	43	27
7	27	47	27
8	32	38	27
Mean	26.63	40.50	26.63
S.D.	5.80	4.14	3.89

APP/PS1/E2-6KApoEp

	RSC	H	EC
1	18	30	15
2	16	31	21
3	13	28	20
4	17	24	19
5	11	27	18
6	25	27	19
7	16	32	15
8	20	33	19
Mean	17.00	29.00	18.25
S.D.	4.28	3.02	2.19

APP/PS1/E3-V

	RSC	H	EC
1	28	45	24
2	24	39	29
3	29	40	25
4	29	41	30
5	38	47	29
6	34	45	32
7	25	36	32
8	29	32	32
Mean	29.50	40.63	29.13
S.D.	4.57	5.04	3.14

APP/PS1/E3-6KApoEp

	RSC	H	EC
1	15	29	15
2	16	20	13
3	20	18	17
4	26	21	13
5	27	28	21
6	20	27	17
7	20	27	26
8	24	19	21
Mean	21.00	23.63	17.88
S.D.	4.38	4.53	4.52

APP/PS1/E4-V

	RSC	H	EC
1	35	61	38
2	39	63	59
3	32	49	41
4	48	58	42
5	42	64	49
6	46	60	36
7	35	79	55
8	36	79	55
Mean	39.13	64.13	46.88
S.D.	5.72	10.26	8.77

APP/PS1/E4-6KApoEp

	RSC	H	EC
1	14	34	23
2	23	33	25
3	26	29	32
4	24	34	21
5	23	31	17
6	22	33	23
7	26	35	24
8	22	38	22
Mean	22.50	33.38	23.38
S.D.	3.78	2.67	4.24

Table S34 (Supporting information of Fig. 6*B*)

**APP/actin ratio**	1	2	3	4	Mean	S.D.
**APP/PS1/E2-V**	1.01	0.99	1.03	0.99	1.00	0.02
**APP/PS1/E3-V**	1.00	0.99	1.01	1.03	1.00	0.02
**APP/PS1/E4-V**	1.05	1.06	1.03	1.06	1.05	0.02
**APP/PS1/E2-6KApoEp**	0.95	0.98	0.93	0.94	0.95	0.02
**APP/PS1/E3-6KApoEp**	0.95	0.94	0.98	0.94	0.95	0.02
**APP/PS1/E4-6KApoEp**	0.98	0.97	1.00	1.00	0.98	0.02

Table S35 (Supporting information of Fig. 7*B*)

**pC99/actin ratio**	1	2	3	4	Mean	S.D.
**APP/PS1/E2-V**	1.05	1.03	1.05	1.02	1.04	0.02
**APP/PS1/E3-V**	1.05	1.02	1.05	1.01	1.03	0.02
**APP/PS1/E4-V**	1.13	1.11	1.14	1.11	1.12	0.02
**APP/PS1/E2-6KApoEp**	0.82	0.78	0.82	0.80	0.81	0.02
**APP/PS1/E3-6KApoEp**	0.82	0.79	0.82	0.78	0.80	0.02
**APP/PS1/E4-6KApoEp**	0.82	0.82	0.83	0.80	0.82	0.01

**C99/actin ratio**	1	2	3	4	Mean	S.D.
**APP/PS1/E2-V**	1.03	1.02	1.05	0.99	1.02	0.02
**APP/PS1/E3-V**	1.03	1.00	1.03	1.00	1.01	0.02
**APP/PS1/E4-V**	1.11	1.09	1.12	1.10	1.10	0.01
**APP/PS1/E2-6KApoEp**	0.77	0.72	0.76	0.73	0.74	0.03
**APP/PS1/E3-6KApoEp**	0.76	0.71	0.74	0.73	0.73	0.02
**APP/PS1/E4-6KApoEp**	0.75	0.80	0.75	0.80	0.77	0.03

Table S36 (Supporting information of Fig. 7*C*)

**Aβ/actin ratio**	1	2	3	4	Mean	S.D.
**APP/PS1/E2-V**	0.97	0.94	0.97	0.93	0.95	0.02
**APP/PS1/E3-V**	0.95	0.92	0.97	0.94	0.94	0.02
**APP/PS1/E4-V**	1.04	1.06	1.08	1.06	1.06	0.01
**APP/PS1/E2-6KApoEp**	0.90	0.87	0.87	0.86	0.87	0.02
**APP/PS1/E3-6KApoEp**	0.89	0.83	0.87	0.86	0.86	0.02
**APP/PS1/E4-6KApoEp**	0.92	0.89	0.89	0.91	0.90	0.02

Table S37 (Supporting information of Fig. 7*E*)

**BACE1/actin ratio**	1	2	3	4	Mean	S.D.
**APP/PS1/E2-V**	1.01	0.99	1.01	0.99	1.00	0.01
**APP/PS1/E3-V**	1.01	0.99	1.01	0.99	1.00	0.01
**APP/PS1/E4-V**	1.02	0.99	1.02	1.00	1.01	0.01
**APP/PS1/E2-6KApoEp**	1.01	0.98	1.01	0.97	0.99	0.02
**APP/PS1/E3-6KApoEp**	1.01	0.99	1.01	1.00	1.00	0.01
**APP/PS1/E4-6KApoEp**	1.02	0.99	1.01	1.00	1.01	0.01

Table S38 (Supporting information of Fig. 8*B*)

**pp44/42/tp44/42 ratio (Upper)**	1	2	3	4	Mean	S.D.
**APP/PS1/E2-V**	0.99	0.95	0.99	0.95	0.97	0.02
**APP/PS1/E3-V**	0.99	0.98	0.96	0.93	0.97	0.03
**APP/PS1/E4-V**	1.01	0.98	1.00	0.98	0.99	0.02
**APP/PS1/E2-6KApoEp**	0.68	0.65	0.67	0.65	0.66	0.02
**APP/PS1/E3-6KApoEp**	0.65	0.62	0.66	0.63	0.64	0.02
**APP/PS1/E4-6KApoEp**	0.61	0.58	0.60	0.58	0.59	0.02

**pp44/42/tp44/42 ratio (Lower)**	1	2	3	4	Mean	S.D.
**APP/PS1/E2-V**	0.93	0.90	0.93	0.90	0.91	0.02
**APP/PS1/E3-V**	0.93	0.90	0.93	0.90	0.92	0.02
**APP/PS1/E4-V**	0.92	0.90	0.93	0.89	0.91	0.02
**APP/PS1/E2-6KApoEp**	0.67	0.63	0.67	0.64	0.65	0.02
**APP/PS1/E3-6KApoEp**	0.63	0.59	0.63	0.59	0.61	0.02
**APP/PS1/E4-6KApoEp**	0.57	0.52	0.57	0.52	0.55	0.02

Table S39 (Supporting information of Fig. 8*C*)

**pp38/tp38 ratio**	1	2	3	4	Mean	S.D.
**APP/PS1/E2-V**	0.84	0.81	0.84	0.81	0.82	0.02
**APP/PS1/E3-V**	0.85	0.81	0.85	0.82	0.83	0.02
**APP/PS1/E4-V**	0.85	0.81	0.85	0.82	0.83	0.02
**APP/PS1/E2-6KApoEp**	0.93	0.90	0.93	0.91	0.92	0.02
**APP/PS1/E3-6KApoEp**	0.93	0.91	0.93	0.91	0.92	0.01
**APP/PS1/E4-6KApoEp**	0.93	0.91	0.93	0.91	0.92	0.01

Table S40 (Supporting information of Fig. 8*D*)

**DLK/actin ratio**	1	2	3	4	Mean	S.D.
**APP/PS1/E2-V**	0.93	0.90	0.94	0.91	0.92	0.02
**APP/PS1/E3-V**	0.93	0.90	0.93	0.91	0.92	0.01
**APP/PS1/E4-V**	0.94	0.91	0.94	0.92	0.93	0.02
**APP/PS1/E2-6KApoEp**	0.83	0.80	0.82	0.80	0.81	0.01
**APP/PS1/E3-6KApoEp**	0.84	0.81	0.84	0.81	0.82	0.01
**APP/PS1/E4-6KApoEp**	0.84	0.82	0.85	0.82	0.83	0.02

Table S41 (Supporting information of Fig. 10*B*)

6KApoEp in brain homogenates	1	2	3	4	Mean	S.D.
**APP/PS1/E2-6KApoEp**	109.91	110.50	112.53	113.12	111.52	1.55
**APP/PS1/E3-6KApoEp**	108.87	112.00	111.26	109.64	110.44	1.44
**APP/PS1/E4-6KApoEp**	112.47	111.66	109.75	113.09	111.74	1.45

A series of 6KApoEp calibration peptides					Mean	S.D.
**6KApoEp (20 ng)**	48.97244	45.19383	45.45362	47.83152	46.86	1.84
**6KApoEp (40 ng)**	143.8317	146.5048	143.9176	148.3046	145.64	2.17
**6KApoEp (80 ng)**	191.6943	188.4303	189.1642	193.3116	190.65	2.26
**6KApoEp (160 ng)**	209.3524	206.3605	208.2171	210.8873	208.70	1.91
**6KApoEp (320 ng)**	217.0475	217.7079	220.1938	220.7631	218.93	1.83

Table S42 (Supporting information of Fig. 10*D*)

**6KApoEp with mouse plasma (h)**	1	2	3	4	Mean	S.D.
**0**	188.97	191.73	193.39	189.92	191.00	1.96
**3**	192.71	191.01	194.80	192.24	192.69	1.58
**6**	190.19	191.52	193.87	190.87	191.61	1.60
**12**	193.65	194.17	191.49	195.13	193.61	1.54
**24**	193.03	196.00	194.41	191.45	193.72	1.94
